# Developing a monitoring and assessment program with diatoms, an improved metric calculation method, and causal analysis for Big Cypress National Preserve, Florida (USA)

**DOI:** 10.1007/s10661-026-15144-0

**Published:** 2026-03-15

**Authors:** R. Jan Stevenson, Kevin R. T. Whelan, Michelle C. Prats

**Affiliations:** 1 Department of Integrative Biology, Michigan State University, East Lansing, MI USA; 2 South Florida/Caribbean Network, National Park Service, Inventory and Monitoring, 18001 Old Cutler Rd. Suite 419, Palmetto Bay, FL 33157 USA

**Keywords:** Algae, Ecology, Management, Metrics, Periphyton, Wetlands, Phosphorous

## Abstract

We used relationships between diatom species composition and phosphorus concentration in periphyton (mat P) to develop a monitoring and assessment program for wetlands in the Big Cypress National Preserve (BCNP) in Florida, USA. Cluster analysis and regression showed limitation of taxonomic composition of assemblages to a few low phosphorus (P) taxa in low mat P conditions, with additional species being able to colonize habitats having higher mat P concentrations. TITAN (Threshold Indicator Taxa Analysis) and regression respectively identified 11 and 8 low P taxa and both methods identified 47 high P taxa. Congruence of low and high P taxa traits determined in our study and results of prior phosphorus experiments confirmed P caused taxon responses. We varied taxa traits (low and high P taxa), method for determining traits, and trait calculation method to evaluate metric performance. Metrics of biological condition, whether using the low or high P taxa traits, were most highly related to mat P when using TITAN derived taxa traits and a novel relative abundance calculation using log-transformed relative abundances. Benchmarks for management targets were determined for minimally disturbed conditions and for greater than usual changes in assemblages along the P gradient. Diatom metrics were more highly correlated with distance from P sources than mat P, indicating species-based metrics have high value for monitoring and assessment. Our results can be used for a monitoring and assessment program with effects-based management targets in BCNP, and our methods provide examples for applications in other ecological settings.

**Supplementary Information:**

The online version contains supplementary material available at 10.1007/s10661-026-15144-0.

## Introduction

Building a thorough assessment program depends on management goals and an understanding of the target ecosystem (Patterson et al., 2008; Stevenson et al., 2004a; USEPA, 1992). Understanding the target ecosystem informs which attributes are the target of management goals, what contaminants and habitat alterations (herein called stressors, sensu USEPA, 1992) affect those management goal attributes, and what human activities produce those stressors (Stevenson, 2011; Tang et al., 2020). Measurements of key direct and indirect variables in the ecosystem associated directly or indirectly with management goals should be used to characterize the ecological condition. Assessment can simply characterize the state of resources without comparison to management criteria (e.g., The Heinz Center, 2002). Assessment can also compare ecosystem state to benchmarks in variables that can be used to determine if ecosystem conditions meet management goals and whether management actions are warranted (Stevenson et al., 2004a). Using causal analysis to diagnose which stressors are most affecting management goal attributes can also be part of assessments, so remedial management actions can be selected (Beyers, 1998; USEPA, 2000; Cormier & Suter, 2008) .

We developed a periphyton monitoring program for Big Cypress National Preserve (BCNP). The overarching goal of the BCNP periphyton monitoring program is to support the United States National Park Service (NPS) mission to preserve the unimpaired condition of natural resources in National Parks (Patterson et al., 2008). Periphyton is a microbial mat community composed of algae (mostly taxa of cyanobacteria, green algae, and diatoms), bacteria, fungi, and microfauna (Browder et al., 1994). Periphyton have been studied extensively in the greater Everglades area, which is located southeast of the BCNP. In the greater Everglades and BCNP, a distinctive calcareous periphyton is observed in areas with minimal human disturbance (Browder et al., 1994; La Hée & Gaiser, 2012). Calcareous periphyton have important ecological functions, respond directly and sensitively to phosphorus pollution, and phosphorus effects on periphyton cascade through the rest of the ecosystem and its biota (Gaiser et al., 2005, 2006; Gleason et al., 1974; Hagerthey et al., 2011; McCormick et al., 1997, 2001). Although both nitrogen and phosphorus can regulate species composition of algae in wetlands (Vymazal et al., 1994; Pan & Stevenson, 1996; Cooper et al., 2016), phosphorus regulates biomass and species composition of calcareous periphyton in the Everglades. For these reasons, periphyton were selected as one of the vital signs for monitoring in BCNP (Patterson et al., 2008) with park-specific goals to determine status and trends in periphyton, water quality, and ecosystem function (Urgelles et al., 2019). Differences in soils, wetland transmissivity, and phosphorus range between the BCNP and the greater Everglades (Jarosewich & Wagner, 1985) were deemed sufficient to warrant development and testing of a periphyton program specifically for BCNP.

Often, taxa traits (such as sensitivity or tolerance to pollution) for a new monitoring program are determined using past research in other regions. Lavoie et al. (2009) showed that diatom taxa indicator traits from Van Dam et al. (1994), largely based on research and application in Europe, were related to pollution indicators in Canada. Tang et al. (2016) successfully used diatom taxa traits developed for the western United States for assessments across the country. However, Potapova & Charles (2002) found regional differences in indicator values across ecoregions in the U.S. Therefore, it seems reasonable to start developing a new program by using taxa trait characterizations from past projects in regions with similar types of human disturbance, pollution, and natural variability. Then, when sufficient data has been accumulated, as in BCNP, refine those diatom taxa traits for the specific region being monitored.

Environmental metrics, hereafter referred to simply as metrics, are quantifiable standardized measurements that assess ecological impacts and can clearly communicate this information. Many types of metrics have been proposed to quantitatively characterize biological conditions, ranging from diversity measurements to indicator species abundances and weighted average models that infer stressor conditions (Stevenson, 2014). Given the goal of the BCNP program, metrics should be assessed for their ability to determine the deviation of the assessed ecosystem from minimally disturbed ecological condition (*sensu* Davies Jackson., 2006; Stoddard et al., 2006). In addition to diversity traits and weighted average models that infer specific stressor levels, measures of the proportions of sensitive native or stressor-tolerant taxa align with standard attributes of biological condition (Davies & Jackson, 2006). These metrics are commonly calculated as either the proportion of taxa or proportion of individuals that are either sensitive or tolerant to a stressor, thereby, respectively, omitting or including weights for relative abundance of individuals.

Some studies show that metrics characterizing the proportion of taxa that are sensitive or tolerant to stressors (i.e., presence/absence of taxa) can perform as well as or better than metrics that include weighting for relative abundances of sensitive and tolerant individuals in samples (Charles et al., 2021; Stevenson et al., 2008b; Wang et al., 2005). The high performance of metrics that measure changes in the proportions of taxa with specific traits seems beneficial because those measurements are readily interpreted as indicators measuring losses in sensitive taxa or gains in tolerant taxa (even though there are problems with accuracy measuring true losses or gains in taxa when minute proportions of the organisms in habitats are observed microscopically). Incorporating differences in relative abundances of individuals of those sensitive and tolerant taxa should improve metric performance compared to simpler presence/absence approaches. Perhaps variability in relative abundance of individuals is so great that it reduces precision of metrics. If that is the case, down-weighting abundances with log-transformations could improve metric performance.

The specific goals of this paper were to develop a periphyton monitoring and assessment program for BCNP and to provide an example of an assessment framework (based on Stevenson et al., (2004a, 2004b)) that can be used in other ecosystems. Ideally, we are looking for a metric that tracks a known stressor, based on a specific trait, and that can be informative to management. First, we characterized changes in biological condition along a known stressor gradient (P in BCNP using ordination and cluster analysis) to understand the ecosystem. Then we compared metrics calculated using different sources of traits characterizations (literature as well as newly calculated traits), different traits (low and high P), and different methods for calculating metrics. Specifically, we tested a novel metric calculation using relative log-transformed abundances to reduce negative effects that high variability in abundant taxa could have on metric precision. Finally, we determined how metrics change along a stressor gradient to inform development of effects-based management targets for both biological condition and stressors (minimally disturbed condition and P in the BCNP). Results of this work should provide high performance metrics for monitoring and assessing periphyton in BCNP as well as benchmarks for triggering management actions.

## Methods

### Study area

**Fig. 1:**
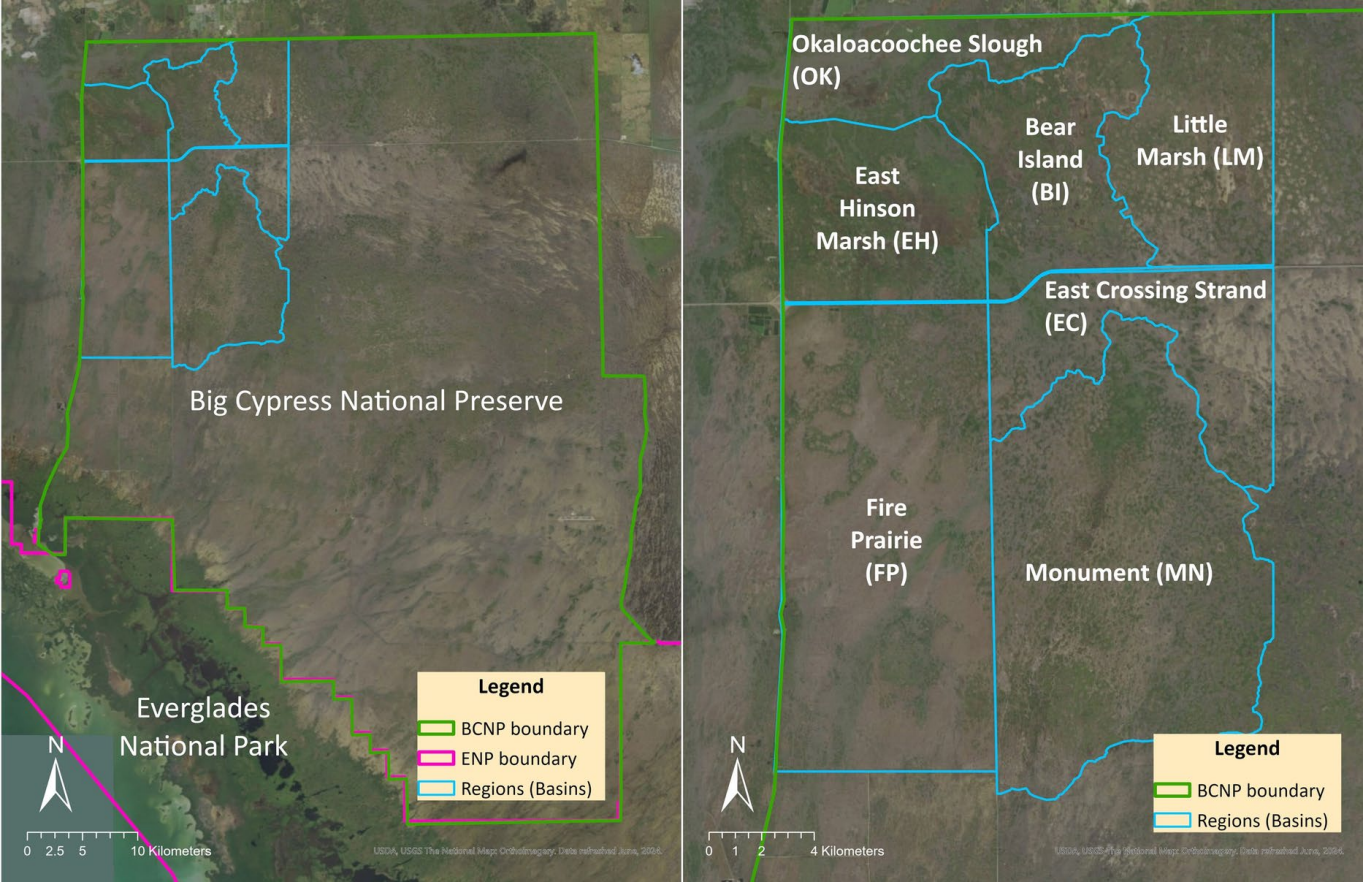
Left panel: Map of BCNP and the study area in the northwest corner. Right panel: Names of regions within the study area

Big Cypress National Preserve is located southwest of Lake Okeechobee and borders the northern edge of Everglades National Park (Fig. 1). It spans 295,016 ha and receives approximately 425,000 visitors annually (Patterson et al., 2008). The preserve contains a large remnant of natural wetland mosaic including cypress strands and domes, pine forests, wet prairies, marshes, sloughs, mangrove forests, and hardwood hammocks. The preserve also contains large stands of dwarf cypress, as well as rare orchids, bromeliads, and ferns (Patterson et al., 2008).

Big Cypress is fundamentally different than its adjacent Everglades neighbor. The soil is comprised of sands (Pamlico Sands) overlying caprock (hardened limestone) of the Tamiami Formation, whereas the vast majority of the greater Everglades system has an organic peat substrate overlying Miami Limestone (Jarosewich & Wagner, 1985). Water transmissivity is more localized and slower in Big Cypress than in the Everglades (Jarosewich & Wagner, 1985). Concentrations of surface water total phosphorus are typically higher in Big Cypress compared to the greater Everglades. This has been attributed to higher total phosphorus from natural sources such as shallow soils, rocks, and ground water (Miller et al., 2004).

The name “Big Cypress” refers to the vast expanse of cypress (*Taxodium distichum* var. *imbricarium* (Nuttall) Croom) rather than to the size of the trees. The larger bald cypress trees were logged during the past two centuries. Extraction of oil, gas, and minerals occurs within BCNP and surrounding areas to the north (Whelan et al., 2020). North of BCNP and south of Lake Okeechobee, agriculture fields have been operating since the early 1900s. To establish the farming industry, the lands were first drained, and the natural water flow was redirected. The western region of Big Cypress has headwaters that begin north of BCNP and are conveyed south via a canal system (Barron River Canal, Miller et al., 2004). Since BCNP establishment in 1974, the city of Immokalee’s population has grown 6 times greater (just north of the Preserve) (US Census data 1980, 2020). In addition, farming and other industrial development have expanded north of the BCNP boundary. The likely high importance of residential runoff as a phosphorus source in BCNP is another contrast with the greater Everglades area, where agricultural runoff is the dominant source (Davis, 1994). The primary management activities have been related to restoration of the human-altered regional hydrology to a more natural flow pattern, improving water quality entering the park from the north, managing invasive species, balancing recreational and extractive uses with long-term sustainability of the system, as well as protecting and preserving natural resources that include rare species (Urgelles et al., 2019).

### Sampling design

Periphyton sampling for this study was conducted from 2013 to 2020 to test methods for using periphyton to assess and track ecological conditions in BCNP. There were two sampling designs used over this period, one for the 2013 and 2014 sampling and one design for the 2017–2020 sampling. The change in sample design was due to an increase in financial allocation to the monitoring program allowing for more helicopter flight time. For both sample designs, the target sampling population was periphyton within mapped graminoid and broadleaf marshes in northwestern BCNP. The sampling frame was restricted to mapped and accessible marshes (which includes access by helicopter, ORV, truck, or hiking) in the northwest section of BCNP (Fig. 1), because human disturbance is adjacent to that section of BCNP. That disturbance produces a gradient from high to low phosphorus concentrations, with minimally disturbed conditions found in the southern and eastern parts of the sampling area.

The northwestern BCNP sampling area is divided into basins separated by artificial or natural structures that may or may not impede water flow during part or all of the year (Fig. 1). Each basin is treated as a separate sampling strata or block. All potential habitats (graminoid and broadleaf marshes) were identified in each basin from the Western Big Cypress National Preserve Vegetation Map (Whelan et al., 2020). Using a restricted stratified random design, 5–7 sites were selected for potential sampling in each of the 7 basins. Site selections were based on the centroid of the map grid cells. A boundary for potential collection locations at each site was drawn with a 250 m radius from the centroid. Each potential site was first evaluated and those meeting specific criteria were sampled annually (Londoño, 2019). Sites were selected once. The goal was to sample the same sites annually (Urgelles et al., 2019); however, annual variations in conditions often required sampling alternate sites. For 2013–2014 samplings, sample design followed similar criteria as above except some sites were haphazardly selected to allow for the following: (a) maximizing spatial spread within a basin, (b) co-location with water-quality monitoring stations where available, (c) answering specific monitoring questions and (d) ensuring half the sites were accessible via helicopter and the other half of the sites accessible via ORV trails.

In general, at least 6 sites were sampled per basin, with there being seven different basins, except in 2013 when there were eight basins. Selected sites within basins were treated as replicates for characterizing status and trends in basin conditions. The number of sites collected each hydroyear were as follows: 49 in 2013; 56 in 2014; 45 in 2017; 59 in 2019; and 49 in 2020. Over the 2013–2020 period, there were 113 unique sites; not all sites were sampled each year. The two general sampling designs resulted in 64 unique sites from 2013 to 2014 and 49 unique sites from 2017 to 2020.

### Field collection methods

Sampling followed a hydrological clock. It was conducted approximately two months after the peak of the wet season (typically November or December). Water was usually present at all sites during collection. However, if the site was dry, then dry periphyton was collected, preserved, and processed the same as wet samples. Attempts were made to access the same depressional marsh for revisits year after year.

Upon arrival, the site was surveyed to locate where water was present for collecting a periphyton sample if possible. A sample consisted of a minimum of five grabs within a 5-m radius sample location, with each grab being split between two 125 ml Nalgene® opaque bottles; one for diatom analysis and one for mat P (phosphorus concentration in periphyton) analysis. Mat P concentrations vary less than water column P as indicators of P pollution and have thus been recommended for the Everglades (Gaiser et al., 2004). The grab samples were typically collected from one substrate and the preferred order of substrate collection was (1) floating mat, (2) algae on plants (sweaters), (3) algae on sediments (benthic mats), and (4) algae on woody debris. Additional site characterization information was collected including vegetation data, pH, water conductivity, and water depth. Samples were preserved as quickly as possible upon return from the field. Periphyton mat P samples were chilled in an ice slurry in the field and frozen at the lab. The periphyton diatom samples were fixed with a 3% buffered formalin solution (Muxo & Shamblin, 2019). Excess water was decanted prior to fixation.

### Sample processing and analysis

Periphyton samples were sent to Florida International University for the assay of mat P, which involved digesting mats and assaying total phosphorus (TP). In the laboratory, periphyton sample wet weights were recorded, thawed, and then extraneous plant material and animals were manually separated from the periphyton and placed in foil packets. These were placed in an 80 °C oven for 3 days; once dry, the weight was recorded as “extraneous material,” and then discarded. The TP content of periphyton was expressed on a dry-weight basis because the organically incorporated P was not separable from that bound to calcite. The TP subsample was dried at 80 °C and ground down to a fine powder with a mortar and pestle. Colorimetric analysis was used to estimate TP concentrations of the periphyton subsamples following the methods of Solorzano and Sharp (1980), which were then used to calculate µg g^−1^ dried periphyton mat (not ash free dry mass). For hydroyears 2013, 2014, and 2017, the lab methods followed Smith (2018), and for hydrologic year 2019 and 2020, they followed Wilson (2020). The difference in methods is due to the acquisition of a newer spectrophotometer.

Early, unpublished analyses of data indicated diatom species composition was related to the human disturbance gradient in BCNP as well as the composition of both diatom and non-diatom algae. Afterward, algal analyses were limited to diatom species composition for the development of diatom metrics to reduce the costs of periphyton analyses. Periphyton samples were sent to Michigan State University to be assayed for diatom species composition. There, samples were acid cleaned in nitric acid and mounted on microscope slides in NAPHRAX^©^. At least 600 diatom valves were identified and counted for each slide. Photomicrographs were taken of all taxa with proportional relative abundance greater than 0.05 in any sample and then archived in the National Park Service database for future reference.

### Data analysis: data selection and descriptive analyses

We created independent calibration and validation datasets with only one sample per site in each dataset to avoid problems with pseudoreplication and repeated measures. The calibration dataset was created using samples from sites having matching mat P samples from the 2013–2020 period by randomly selecting one sample from each site without replacement of selected sites. This created a calibration dataset with 113 samples, each from a separate site, for characterizing changes in diatom assemblages along the P gradient and for characterizing species traits. Of the 113 samples in the calibration dataset, only 78 had all the chemistry information. This reduced 78-sample calibration dataset was used for analyses needing chemistry data.

We restricted development of the validation dataset for metric testing to hydrologic years 2017, 2019, and 2020 when the same taxonomist counted all samples. The validation dataset had 44 samples across 7 regions with one sample per site for either 2017, 2019, or 2020. The rationale for restricting sample selection to the same taxonomist is detailed in the Supplemental Information. In summary, taxonomist and environmental factors varied among years as did residuals in metric-mat P relationships. So we wanted to ensure variability due to the taxonomist did not affect our assessments of metric performance, which is likely the case based on results presented in the Supplementary Information.

Relationships between species composition and environmental factors were evaluated with ordination and cluster analysis using the calibration dataset. Non-metric multidimensional scaling (NMDS) was selected to ordinate samples in species space and then relate environmental variables to the NMDS axes with the *vegan* package in R (Oksanen et al., 2020). Cluster analyses using Bray-Curtis dissimilarity and the calibration dataset were calculated with the *clust* command in R to observe patterns in species composition without some regression-related constraints of ordination. Groups of sites with low dissimilarity were identified and then differences in mat P, conductivity, and pH among these site groups were determined.

To more thoroughly understand and illustrate the succession in diatom assemblages along the P gradient, we used a stacked bar chart using the most abundant low and high P indicator species. Relative rates of change in species relative abundances and the dominance of these taxa in assemblages along the P gradient were determined for the calibration dataset. The most abundant low P and high P taxa were selected for the figure. In addition, we created a heat map of all taxa in 5 samples or more using an Excel^©^ spreadsheet. The heat map matrix had taxa abundances colored in rows and samples in columns, with rows ordered by TWINSPAN groups (R package) and columns ordered by increasing mat P concentrations. Cells of the heat map matrix were colored yellow, orange, or red to indicate increasing relative abundances of taxa.

### Data analysis: taxa trait characterization

Taxa have been characterized by the NPS as being characteristic of oligotrophic or eutrophic habitats based on consensus and best professional interpretation of information reported in literature sources (Table S1). This literature review assessed 13 papers and attempted to determine the optimum TP, trophic habitat preference, pH preference, and trophic indication status of diatoms at the species level.

Traits analysis was conducted to characterize taxa as low and high P taxa using the calibration dataset. Multiple trait characterization methods were used to be thorough and to evaluate consistency in characterizations. Linear and polynomial regression were used to determine whether relationships between taxa relative abundances and mat P concentration were negative or positive to identify low and high P taxa, respectively. Polynomial regression was used to complement linear regression to ensure non-linear responses were detected. TITAN (Threshold Indicator Taxa Analysis (Baker & King, 2010)) was also used to describe taxa with decreasing and increasing indicator species values along the TP gradient for low and high P taxa, respectively. An indicator species value characterizes the association of a taxon with one group of samples versus another and accounts for both occurrence frequency and relative abundance of a taxon (Dufrȇne & Legendre, 1997). TITAN calculates indicator species values with two sample groups separated along a continuous variable and finds the level of that continuous variable that provides the greatest indicator values. TITAN then standardizes the indicator value scores as a z statistic and determines whether the indicator values increase (z +) or decrease (z−) with increasing values of the continuous variable (Baker & King, 2010). Finally, weighted average TP optima were calculated for taxa. Low and high P characterizations (i.e., taxa traits) were only calculated for taxa observed in 5 or more samples from the calibration set of 78 samples from different sites and having P concentrations measured because TITAN requires 5 or more observations per taxon to provide reasonable statistical power for characterizing taxa traits with regression. Mat P optima and tolerances were determined for all taxa because our experience showed weighted average models (WAM) were most precise when all taxa are used in the model. The NPS does not plan to use the WAM model in future monitoring, but it was used here to provide a benchmark for a model that is usually the most precisely related to an environmental gradient (Reavie et al., 2008).

Taxa traits determined with the 78-sample calibration dataset were compared to taxa traits observed in past Everglades research to compare consistency in results and evaluate causal relationships between low and high P traits and P concentrations. Pairwise relationships between taxa trait characterizations in this study and those by Hagerthey et al. (2012) and Gaiser et al. (2006) were determined. Species characterizations for BCNP were also compared to low and high P characterizations by Slate and Stevenson (2007), which were determined by experimental manipulation of P with in situ mesocosms. Identifications of taxa in Gaiser et al. as well as Slate and Stevenson could be related to those in our study because we have been involved with a taxonomic harmonization program with the Gaiser et al. team using photomicrographs of taxa and have the Slate and Stevenson plates of taxa images. For Hagerthey et al., we matched taxa names and accounted for updates in taxa names based on taxonomic literature over the past 20 years. Comparisons with experiments enabled establishing a causal relationship between P concentration and relative abundances of diatom taxa (Beyers, 1998).

### Data analysis: metric development and testing

We then evaluated metric design on their performance to be sensitive to environmental change, to characterize changes in biological condition along the human disturbance gradient, and to help justify management targets for P concentration. We did not expect the same metric to be best for all goals (Fig. S1, Stevenson, 2006, 2011). We sought a metric with a linear response to P concentration to be sensitive to environmental change because metrics with linear response have the same sensitivity to incremental changes in P concentration at all levels of P concentration. We sought non-linear responses of metrics of biological condition to detect thresholds in biological responses that could be used to justify P management targets. We did not calculate a multimetric index because that was not the plan for BCNP application.

For each sample we calculated multiple metrics with traits plus three diversity metrics: taxa number observed in standardized counts, Shannon diversity, and Pielou’s evenness. Counts were standardized to 600 valves because some counts had more than 600 valves. Counts were standardized by assigning random numbers to valves in counts and picking valves with the lowest 600 numbers.

Twenty-four metrics were calculated for each sample, by varying traits (low and high P), having three sources of information for the traits, and four ways to calculate metrics. For each trait, four metric types were calculated: (1) proportion of individuals with a trait (PropValves) as sum(v_ijt_/V_j_), where v_ijt_ is the number of valves counted for taxon *i* with trait *t* (e.g., either high or low P using a specific trait determination method) in sample *j*, and *V*_j_ is the number of valves of all taxa with assigned traits in sample *j*; (2) the proportion of taxa with a trait (PropTaxa), as *t*_tj_/*T*_j_ where *t*_tj_ is the number of taxa with trait *t* in sample *j* and *T*_j_ is the number of taxa with traits assigned in sample *j*; and (3) the number of taxa in a sample with a specific trait (noTaxa, e.g., number of low P taxa). The fourth metric type was RlogA and is described in the next paragraph. Below we also describe our use of the three trait determination methods (i.e., sources): review of the literature, TITAN, and regression.

Recent papers have shown metrics calculated as proportion of taxa with a trait are often related better to human disturbance and stressor gradients than metrics calculated as proportion of individuals with a trait (Carlisle et al., 2022; Stevenson et al., 2008b). Because relative performance of species in a habitat likely varies with environmental conditions as well as their presence in a habitat, we tried down-weighting metrics by log-transforming relative abundances of taxa and thereby reducing variability caused by slight changes in growth rates of abundant taxa that could produce great differences in a taxon’s abundance. We determined relative log abundance (RlogA_ijt_) by dividing the natural log of valve numbers of taxon *i* with trait *t* in sample *j* (log(A_ijt_)) by the sum of natural log transformed valve numbers for all taxa with traits assigned in sample *j* (sum(log(A_ij_)). For each trait determination method, a sample’s RlogA_jt_ metric value for either high or low P RlogA_jt_ was calculated as the sum of all RlogA_ijt_ for taxa with either low (*t* = 1) or high P (*t* = 2) traits divided by the sum of all RlogA_ijt_ for taxa with traits assigned (both low and high P traits), i.e., RlogA_jt_ = sum(RlogA_ijt_)/sum(RlogA_ij_).

Metrics were evaluated by comparing their adjusted *R*^*2*^ values determined for slopes in relationships between metrics and log-transformed mat P. We log_2_ transformed mat P to even the distribution of the observations along the mat P gradient, because the number of low mat P samples was much greater than the number of high mat P samples. Unless described otherwise, a metric-mat P relationship in the following text refers to the relationship between a metric and log-transformed mat P. The R package *lm* was used for linear regression (R Core Team, 2021).

Both actual values and ranks of *R*^*2*^ values were evaluated. Three analyses of variance (ANOVA) were used to compare average adjusted *R*^*2*^ for metric-mat P relationships for the following: (1) different metric types (noTaxa, PropTaxa, PropValves, RlogA); (2) trait source (literature, regression, or TITAN); or (3) trait (low P versus high P).

To control for other metric attributes (metric type, trait source, and trait) when evaluating performance of one of the metric attributes, Kruskal tests (*kruskal.test* in R) were run to compare ranks of adjusted *R*^*2*^ of metric-mat P relationships. We use the Kruskal tests to determine whether performance statistics for multiple metric-mat P relationships, in this case adjusted *R*^*2*^, are consistently higher using one method or another. Rather than simply comparing the relative number of metric-mat P relationships in a large table that are higher using one calculation method than another, non-parametric Kruskal tests allow us to more rigorously determine the probability that a comparison of ranks of performance statistics could occur by chance. Because the organization of the comparison of metric performance methods is rather complicated, we describe them in detail in the Supplemental information.

Mat P and metrics were related to latitude (lat) and longitude (long) to determine their relationship to distance from the main source of human disturbance in the northwestern corner of the BCNP study region. Linear regression (*lm* in R) was used to relate mat P and metrics to latitude, longitude, and a lat-long interaction term.

We also evaluated metrics by determining their precision for distinguishing differences among regions and the confounding effects of natural factors (e.g., water depth and substrate type) on metric-mat P relationships. The methods for these analyses and results are presented in the Supplementary Information.

### Data analysis: management benchmarks

We used multiple lines of evidence to establish benchmarks for assessment and possible management targets for mat P and periphyton metrics. Here we use the term benchmark to mean a level of an environmental variable that could be used by resource managers to set management targets. Non-linear attributes of metrics can be used to develop consensus for management targets (Muradian, 2001). Tipping points in relationships (deviations from the linear patterns) showing assimilative capacity can provide justification for protecting ecosystems at pollution levels below those tipping points and thus preventing major changes in ecosystems (Soranno et al., 2008; Stevenson et al., 2008a; Stevenson, 2011; Fig. S1), such as threshold changes in calcareous periphyton abundance in the Everglades (Stevenson, 2014; Stevenson et al., 2002). We were particularly interested in tipping points at the low-P end of a mat P range, thereby showing natural assimilative capacity for the biological condition attributes measured by a metric (Fig. S1).

We used three approaches for determining benchmarks in biological response along the mat P gradient. Decision tree analysis was calculated relating the 29 trait-based, diversity, and WAM metrics to mat P using the *rpart* package in R (Therneau & Atkinson, 2025) to determine changepoints along the mat P gradient in trees with the lowest error variance. Decision tree analysis finds a breakpoint in the independent variable that groups the data with group means that minimize variation within the groups above and below the breakpoint. We also used the 75th percentile of mat P concentrations for low P groups of samples identified with cluster analysis as another benchmark. We also used the point with highest sum of TITAN *z* scores for low P taxa as a management benchmark (Baker et al. 2010). We compared benchmarks to changes in species composition illustrated in the heat map of taxon abundances in samples with successively higher mat P to interpret biological responses associated with these mat P benchmarks.

We related mat P to latitude and longitude to determine if it was related to distance from the northwest corner of the BCNP study area where human disturbance was highest. To ensure management targets based on effects of mat P did not under or over-protect BCNP wetlands, we determined likely mat P concentrations at sites we assumed were minimally disturbed sites. We selected all sites in the Fire Prairie (FP), Monument (MN), and Little Marsh (LM) regions, even though some sites were close to canals and trails, because these regions were furthest from large-scale human disturbance northwest of the BCNP study area.

## Results

### Changes in diatom assemblages along the P gradient

NMDS with all samples with all chemistries in the calibration dataset (*n* = 78) showed diatom assemblages responded most to mat P. The NMDS had an insignificant deviation from 1 in the stress plot. The continuous variables mat P, year sampled, pH, water temperature, and conductivity as well as the categorical variables habitat, water color, and taxonomist were significantly related to NMDS axes (*p* ≤ 0.041, Fig. S2). Mat P, sampling year, and habitat were the most highly correlated abiotic variables with 0.624, 0.349, and 0.273 *R*^2^ values, respectively (*p* < 0.001, Table S2). pH and water temperature were next most closely related to NMDS axes, with 0.162 and 0.128 *R*^*2*^ values respectively. Mat P and pH were inversely correlated to each other (Pearson *r* = −0.357) along the NMDS axes.

**Fig. 2:**
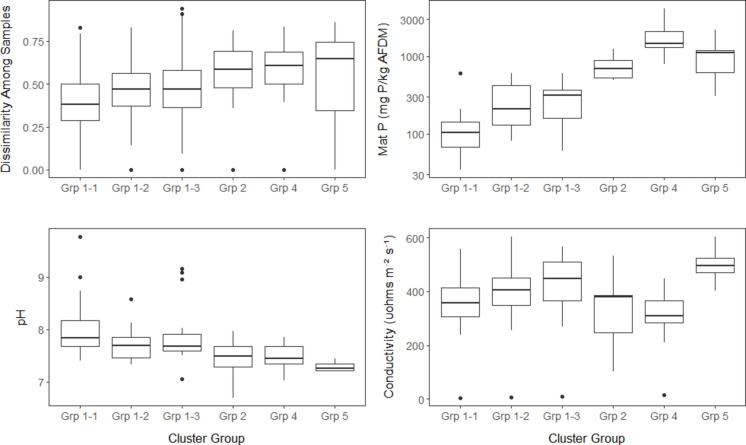
Boxplots of dissimilarity, mat P, pH, and conductivity for the groups of sites delineated in cluster analysis of the calibration data set

Cluster analysis of all 113 samples in the calibration dataset isolated 5 groups of samples from different sites at a dissimilarity level of 0.75 (Fig. S3). From low to high along the mat P scale, these groups were designated 1 to 5. Group 1 had more than two thirds of the samples. Groups 2 to 5 had 5, 1, 9, and 10 samples, respectively. Group 3 was dropped from the following discussion because it only had one sample. Three subgroups in Group 1 that had dissimilarity less than 0.6 among samples were identified and designated as Groups 1–1, 1–2, and 1–3 in order of dissimilarity among groups. Group 1–1 had very low dissimilarity among samples, and Group 1–3 had the highest dissimilarity of the Group 1 subgroups. Dissimilarity increased significantly (Tukey HSD, adj *p* < 0.001, Fig. 2) from a mean of 0.39 among samples in Group 1–1 to 0.46 and 0.47 in Groups 1–2 and 1–3, which were themselves not statistically different. Dissimilarity also increased significantly (Tukey HSD, adj *p* < 0.001) from Groups 1–2 and 1–3 to Groups 2, 4, and 5, which had 0.54 average dissimilarity for those three groups of samples representing sites.

Mat P, pH, and conductivity varied significantly among sites represented by sample groups (ANOVA, *p* < 0.05, Fig. 2). With 75th and 90th percentiles of 142 and 201 µg P g^−1^ dry mass, mat P was lower in Group 1–1 than all other sample groups (Tukey HSD, adj *p* < 0.001). Average mat P for sites with samples in either Group 1–2 or 1–3 was lower than in Group 2, 4, or 5. The 75th and 90th percentiles of all sites with samples in Group 1 were 354 and 508 µg g^−1^ mat P. pH of Group 1–1 sites was greater than sites in either Group 2, 4, or 5 (Tukey HSD, adj *p* = 0.03–0.6). Average conductivity for sites in Group 4 was less than in Groups 1–3 and 5 (Tukey HSD, adj *p* = 0.04). Tukey HSD comparisons of mean chemistry values for pairwise comparisons of other site groups were not significantly different.

**Fig. 3:**
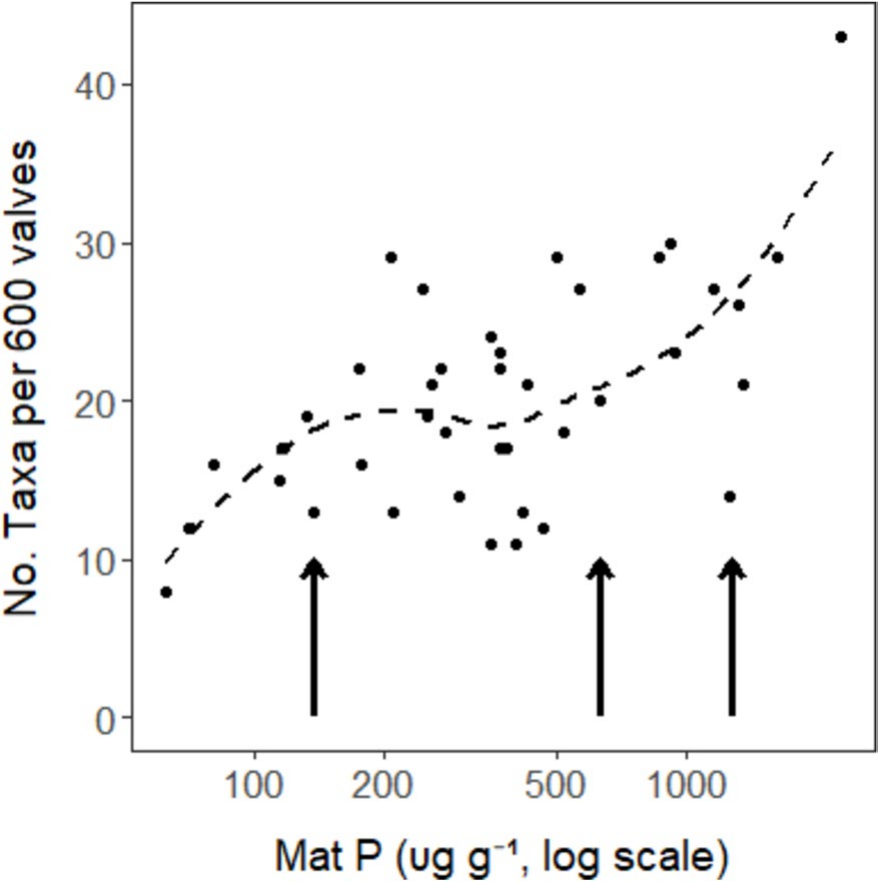
Relationship between species richness and log mat P illustrated by lowess fit line. Levels of mat P associated with changepoints having the lowest probability of occurring by chance are shown by vertical arrows (Table S9)

**Fig. 4:**
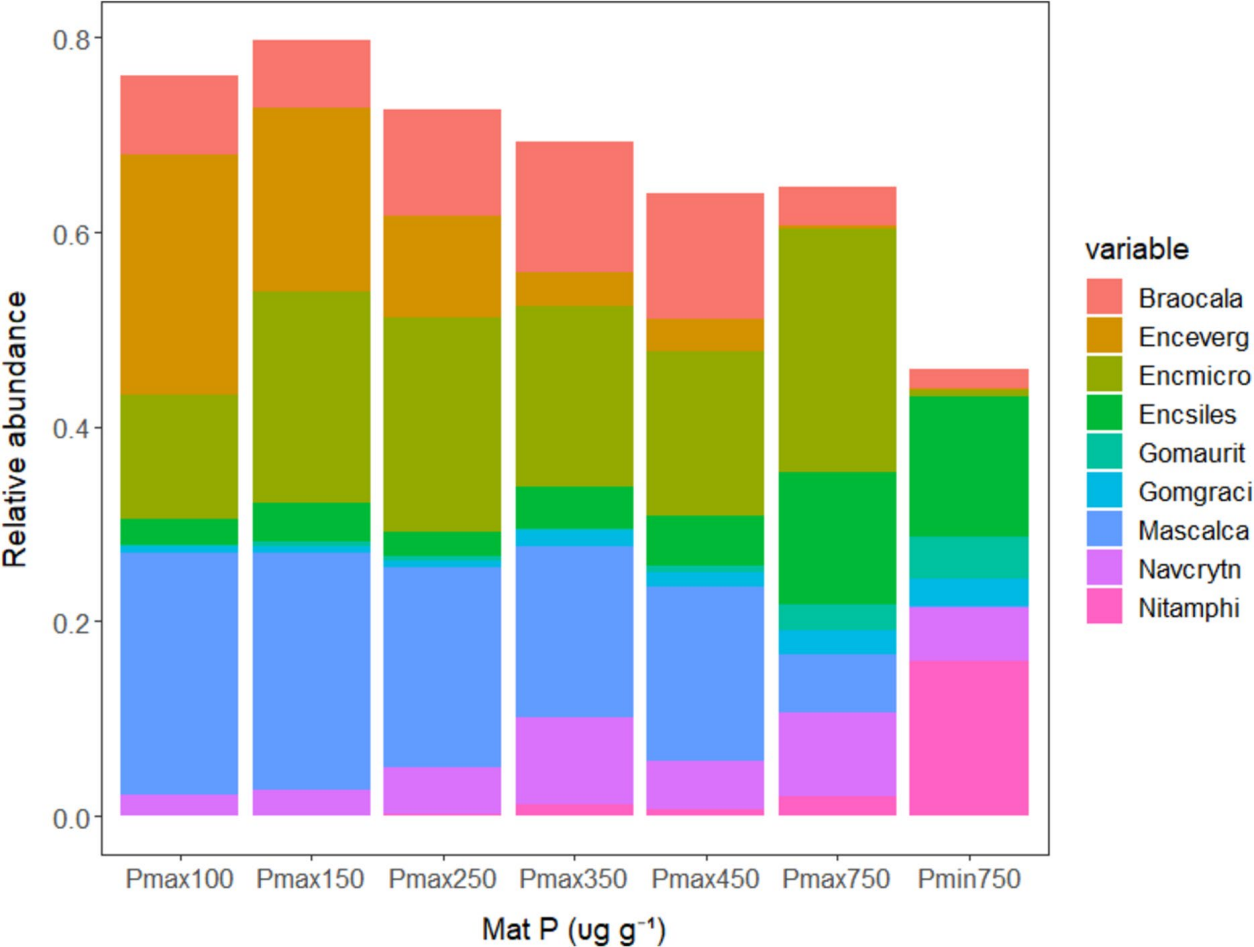
Average relative abundances of dominant species in groups of samples with increasing mat P concentrations illustrated with a stacked bar chart. Braocala *Brachysira ocalanensis*, Enceverg *Encyonema evergladianum*, Encmicro *Encyonema microcephala*, Encsiles *Encyonema silesiacum*, Gomaurit *Gomphonema auritum*, Mascalca *Mastogloia calcarea*, Navcrytn *Navicula cryptotenella*, and Nitamphi *Nitzschia amphibia*

The number of diatom taxa observed in 600 valve counts increased (*p* < 0.001) from about 10 in low P to 25 in high P as high P taxa colonized and low P taxa became very rare or were lost (Figs. 3, 4, and S4). *Encyonema evergladianum*, *Mastogloia calcarea, Encyonopsis microcephala*, and *Brachysira ocalanensis,* assigned low P traits in calculations described later in these results, comprised approximately 65% of assemblages in low mat P conditions. Their combined proportion of assemblages decreased to 45, 20 and 5% in samples with mat P ranging, respectively, from 350–450, 450–750, and greater than 750 µg g^−1^ mat (Fig. 4). The five dominant high P taxa *(Encyonema silesiacum, Navicula cryptotenella, Nitzschia amphibia, Gomphonema gracilis, and Gomphonema auritum*) increased from 6% when mat P was less than 100 to about 45% of samples when mat P was greater than 750 µg g^−1^ (Fig. 4), leaving a high diversity of other diatom taxa to comprise the rest of assemblages in high P conditions (Fig. S4). Whereas other common low P taxa maintained relatively high proportions of counts at intermediate concentrations of mat P, proportions of *E. evergladianum* decreased more rapidly than other low P taxa (Fig. 4). *Encyonema evergladianum* was observed in 56 of 58 samples with mat P less than 300 µg g^−1^ and were usually quite common; however, it was only observed in 4 of 28 samples in samples with mat P greater than 550 µg g^−1^ (Fig. S4). In contrast, *N. amphibia* was only observed in 6 of 49 samples with mat P less than 200 µg g^−1^, but it was highly abundant in 14 of 16 samples with mat P greater than 800 µg g^−1^.

### Taxa traits

Low and high P traits were assigned to 58 taxa with TITAN and to 55 taxa with regression (Table S3). Weighted average mat P optima were assigned to 157 taxa, which was more than TITAN or regression because the criterion of observing taxa in 5 or more samples was not applied for the determination of mat P optima. Forty-one taxa had low and high P traits assigned from the literature by the NPS. Many more taxa were assigned high P than low P traits. The number of taxa assigned low and high P traits was, respectively, 11 and 47 taxa by TITAN, 8 and 47 taxa by regression, and 23 and 39 by the literature review. TITAN and regression did not assign different traits, either low P or high P, to any taxon, but some of the less common taxa were assigned traits by only TITAN or regression. Low and high P trait assignments did differ for one taxon, *E. silesiacum*, which was assigned a high P trait by TITAN and regression and a low P trait using literature references. Many of the abundant low and high P taxa determined by TITAN and regression did not have trait assignments from the literature. The averages of P optima were, respectively: 179 and 1001 for low and high P TITAN taxa; 187 and 1220 for low and high P regression taxa; and 394 and 1062 for low and high P literature-defined taxa.

*Encyonema evergladianum, M. calcarea, E. microcephala,* and *B. ocalanensis* were the four most common low P taxa (Table S3, Fig. 4). The other four taxa classified as low P by TITAN as well as regression were an unknown species of *Nitzschia* (sp. 1*), Fragilaria synegrotesca, Adlafia bryophila*, and *Nitzschia serpentiraphe*. The most common high P taxa were *E. silesiacum, N. cryptotenella, N. amphibia, G. auritum,* and *G. gracile* (Table S3). These most common taxa were not necessarily the most limited to high and low P conditions. The relative preference of taxa for low and high P conditions, versus the absolute preference (low or high), was important for metrics only when calculating the WAM diatom inferred mat P. Of the abundant low P taxa, *Encyonema evergladianum* had the lowest mat P optimum (112 µg g^−1^, Table S3). *Nitzschia amphibia* had one of the highest mat P optima of the common high P taxa (1198 µg g^−1^). *Encyonema silesiacum* and *N. cryptotenella* had relatively low P optima for taxa characterized by TITAN and regression as high P taxa (439 and 358 µg g^−1^ respectively).

Traits assigned in this project with BCNP samples were highly correlated with traits assigned to taxa in three other studies (Fig. S5). Taxalists did vary between our BCNP list and lists in Hagerthey et al. (2012), Gaiser et al. (2006), and Slate and Stevenson (2007). We felt confident comparing BCNP traits for 35 taxa with names that were clearly the same with Hagerthey et al., 23 taxa in Gaiser et al., and 33 taxa in Slate and Stevenson. Spearman correlations among trait values in paired taxalists were positive, were all highly significant (*p* < 0.001), and had *R*^2^ 0.676, 0.737, and 0.783 for Hagerthey et al., Gaiser et al., and Slate and Stevenson, respectively.

### Metric testing and evaluation: metric-mat P relationships

**Fig. 5:**
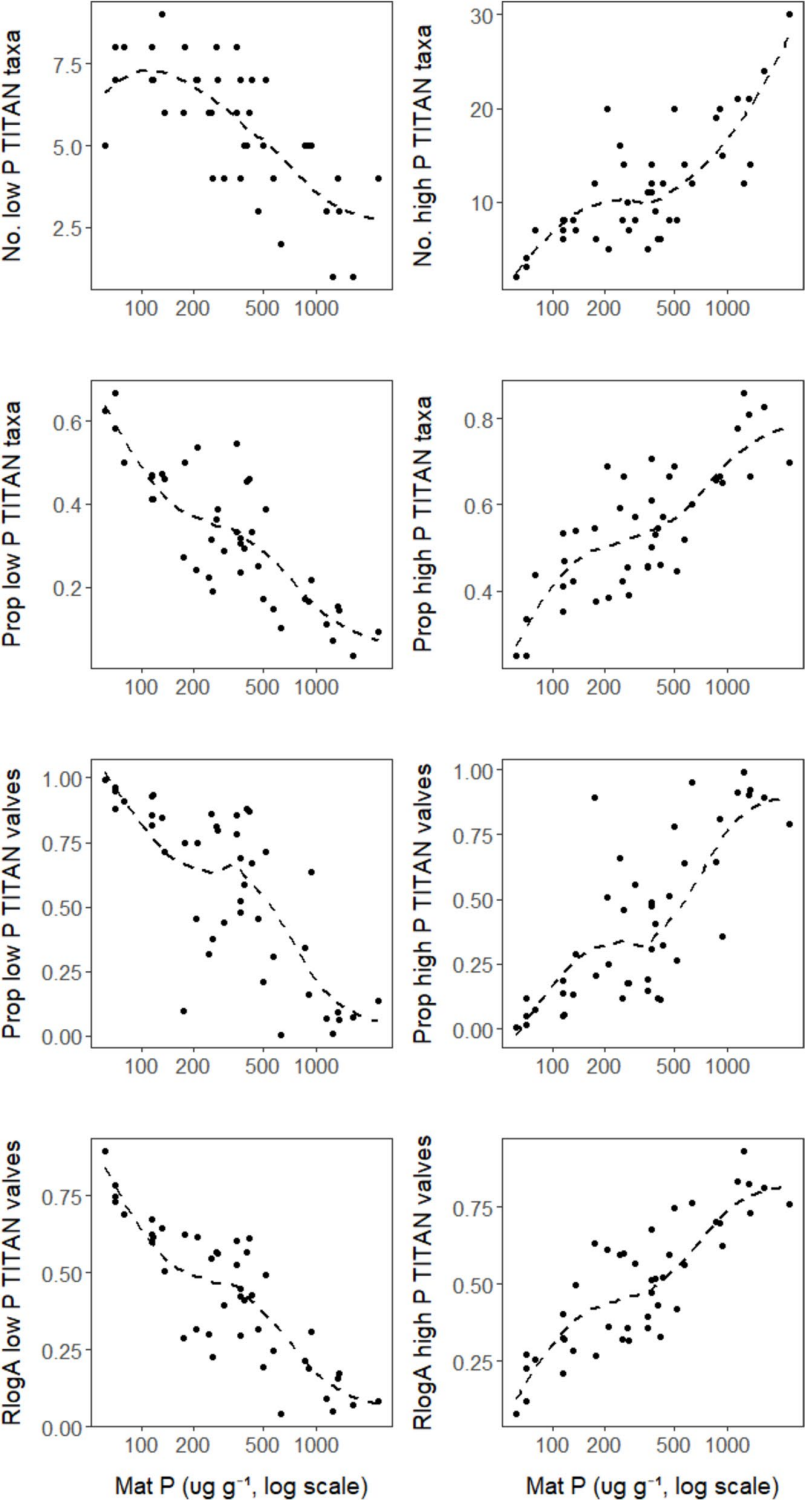
Relationship between TITAN trait metrics and log mat P. The dashed line is a lowess fit to the data. The metric-mat P relationships for all other metrics are shown in Figs. S8 and S9

**Fig. 6:**
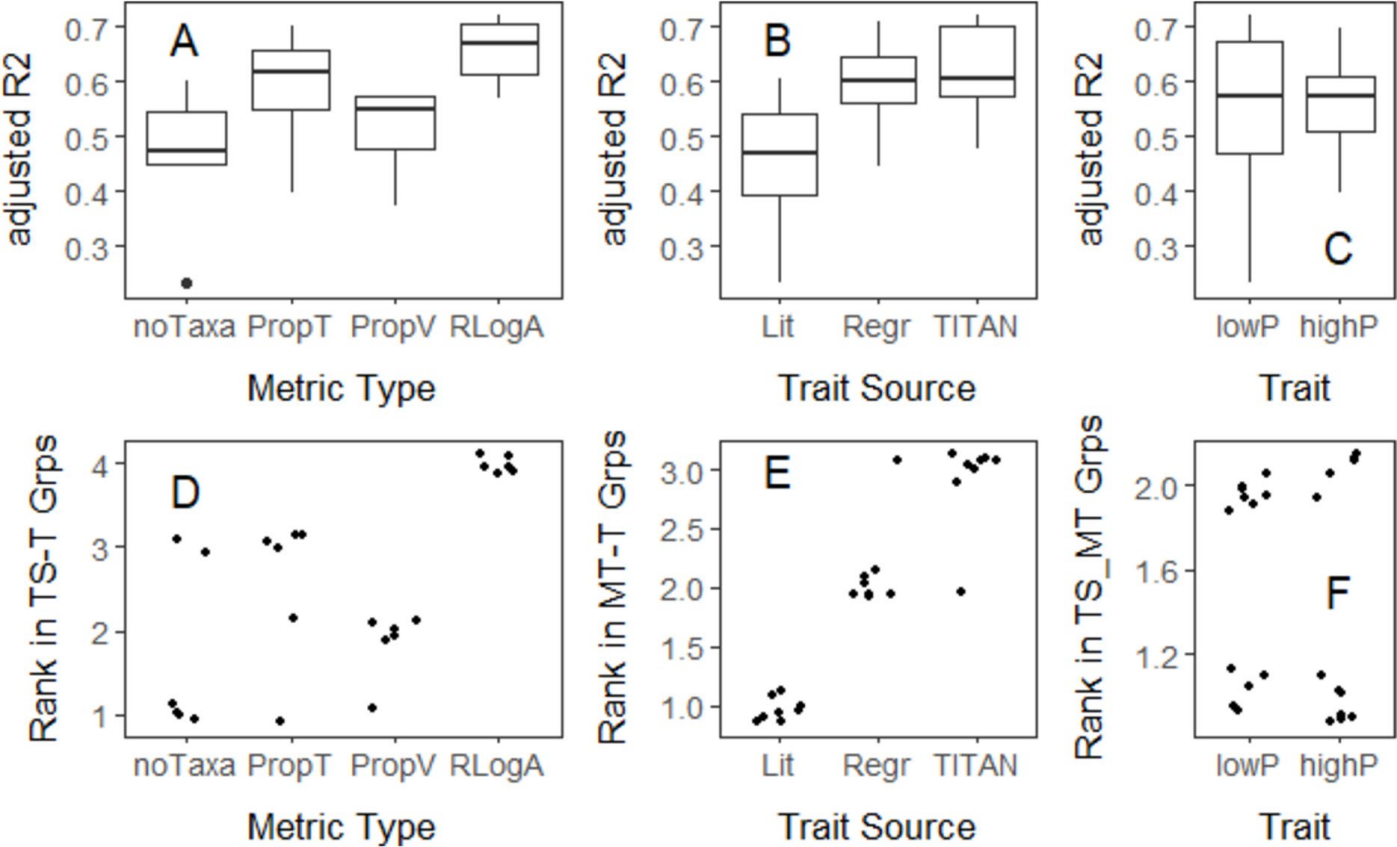
Adjusted *R*^2^ and ranks of metric performance for metrics with different traits. **A**–**C** Effects of metric type, trait source, and trait on adj. *R*^2^ of metric-mat P relationships. **D**–**F** The ranks of each adj. *R*^2^ of metric-mat P relationships to show effects of metric type, trait source, and trait on metric-mat P relationships, using rank of metric-mat P relationships for metric type (ranks 1–4) within trait-source-trait groups, for trait source (ranks 1–3) within metric type-trait groups, and trait (ranks 1–2) within trait source-metric type groups

Almost all metrics except the diversity metrics (Shannon H and Pielou’s evenness) were related to mat P with high statistical significance and adjusted *R*^*2*^ (Table S4). When comparing metric performance, metrics calculated with the relative log abundances (RlogA) were more precisely related (based on adjusted *R*^*2*^) to the mat P gradient than all other metrics, including WAM diatom inferred TP (Figs. 5 and 6, Table S4). According to ANOVA and Tukey HSD, average adjusted *R*^*2*^ for relationships between RlogA metrics and log_2_(mat P) were significantly higher than the average adjusted *R*^*2*^ for mat P relationships with noTaxa metrics (Fig. 6A, Tukey HSD, *p* = 0.016), but other pairwise comparisons among metric types did not differ (ANOVA, n = 24, *p* = 0.017; Tukey HSD, 0.09 < *p* < 0.83, Fig. 6A). However, the ranks of RlogA metric performances were greater than noTaxa, PropTaxa, and PropValves metrics (Kruskal test, *n* = 24, *p* = 0.001; Fig. 6D; Table S4). Ranks of adjusted *R*^*2*^ values for relationships between RlogA metrics and log_2_(mat P) were higher than all other 18 metric relationships with mat P (Fig. 6D). Using the same ANOVA tests of adjusted *R*^*2*^ and Kruskal tests for ranks of metric-mat P relationships, TITAN-based metrics performed better than metrics based on literature or regression traits. Averages for adjusted *R*^*2*^ for metric-mat P relationships, as well as their ranks, were lower for Lit than TITAN and regression sources of traits (Fig. 6B and E, Tukey HSD *p* < 0.026, Kruskal test *p* < 0.0001). Although there was great overlap in adjusted *R*^*2*^ values of metric-mat P relationships based on traits determined by TITAN and regression (Fig. 6B), for 7 out of 8 comparisons, TITAN traits had higher ranks than regression traits for adjusted R^2^ (*p* = 0.008, Fig. 6E). Neither ANOVA nor Kruskal tests indicated differences in performance of low and high P metrics (Figs. 6C, 6 F).

As with metric-mat P relationships, all metrics except the diversity metrics Shannon H and Pielou’s evenness were related with high statistical significance and adjusted *R*^*2*^ to our geographic indicators of human disturbance, latitude, longitude, and the lat-long interaction term (Table S5). In a comparison of which metrics and mat P were most precisely related to the lat-long human disturbance gradient, all metrics using regression and TITAN traits and both WAM metrics had greater adjusted *R*^*2*^ than mat P. Mat P was related to the lat-long model with a 0.44 adjusted *R*^*2*^, whereas the four metrics with highest adjusted *R*^*2*^, which included both WAM metrics, had adjusted *R*^*2*^ ranging from 0.57 to 0.65 (Table S5).

### Precision for distinguishing differences among regions and effects of natural factors

Results describing the precision of metrics for distinguishing differences among regions and effects of natural factors on metrics are presented in the text of the Supplementary Information, as well as in Figs. S6-S7 and Tables S6-S8.

### Management benchmarks

Given that metrics were evaluated along a log-transformed mat P gradient to even distribution of observations along the mat P gradient for regression analysis, that affected linearity of relationships to mat P. Metrics were linearly related to log-transformed mat P according to comparisons of linear and non-linear models (e.g., Figures 5, S8). However, most metrics were non-linearly related to the mat P gradient when mat P was not log-transformed (Fig. S9). With untransformed mat P, metrics with low P taxa traits had a negative exponential relationship with mat P with rapid decreases in low ranges of mat P and little decrease in high ranges of mat P. In contrast, metrics with high P traits increased asymptotically with untransformed mat P with rapid metric increases in the low mat P range and little change in metrics in the high range of mat P.

**Fig. 7:**
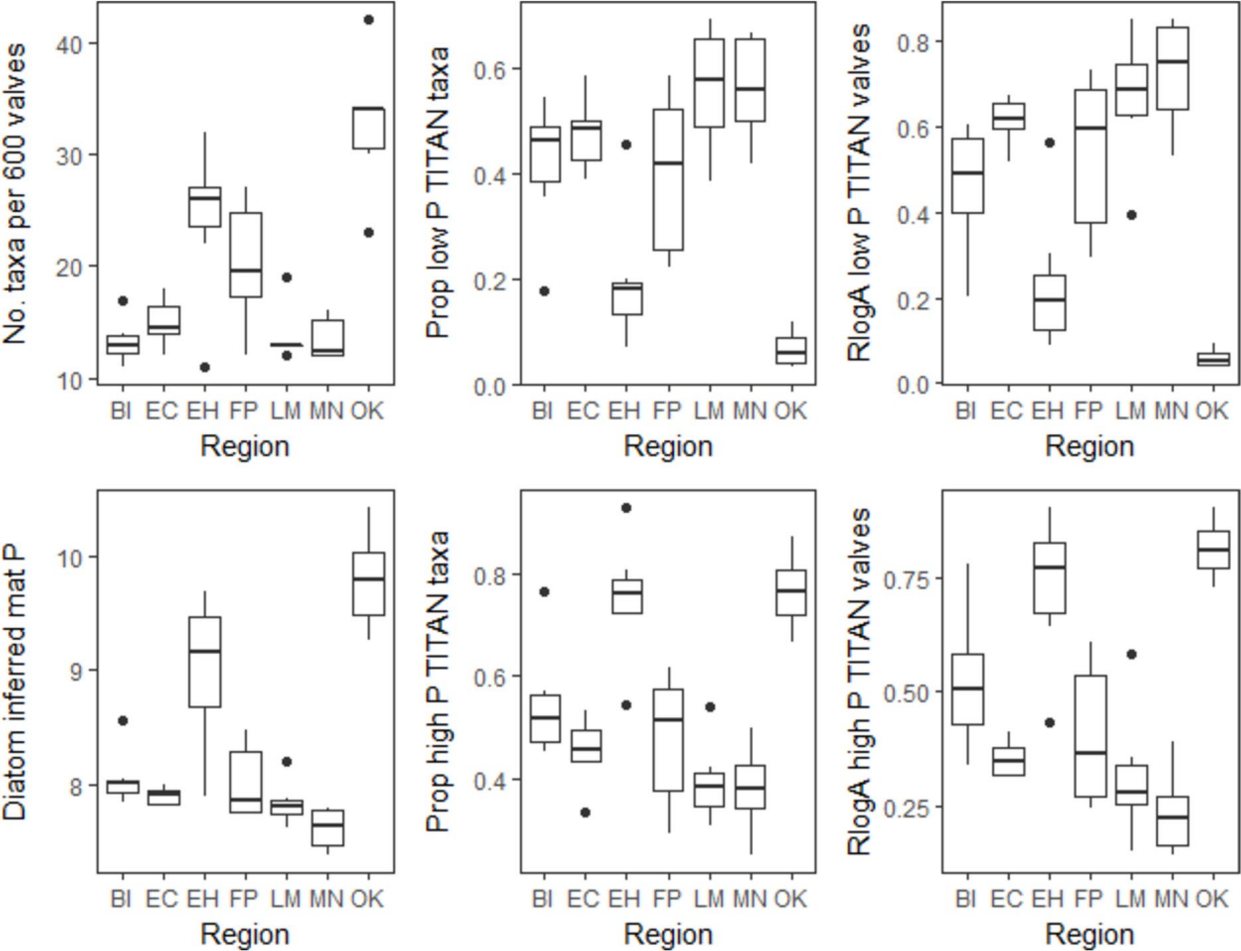
Relationship between number of low and high P TITAN taxa and log mat P with delineations for changepoints determined with decision tree analysis. Levels of mat P associated with changepoints having the lowest probability of occurring by chance are shown by vertical arrows. The lines are a lowess fit to the data

**Fig. 8:**
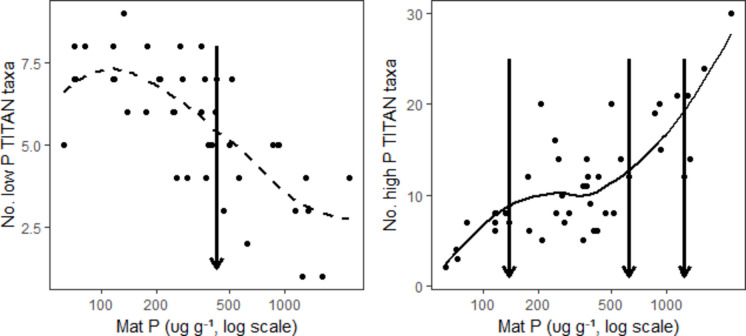
TITAN sum(z−) for low P taxa (filled black circles) and sum(z +) for high P taxa (open circles) for all candidate changepoint along the mat P gradient (labeled environmental gradient). The dashed and solid black lines show the cumulative frequency distribution of changepoints resulting from 500 bootstrapped replicates along the mat P gradient for low P (z−) and high P (z +) taxa, respectively

Benchmarks for relatively sudden changes in taxonomic composition were observed along the mat P gradient for 4 of 13 metrics evaluated. We evaluated mat P relationships with the 3 diversity metrics, 2 WAM metrics, and 8 TITAN-trait metrics. We excluded trait-based metrics that used literature and regression traits from the following analysis to prevent redundancy with TITAN-trait metrics that were best related to log mat P. Changepoint analysis showed that a breakpoint for low P TITAN taxa occurred at 427 µg g^−1^ mat P, above which all low P taxa numbers were low (Fig. 7, Table S9). Changepoint analysis showed numerous statistically significant (*p* ≤ 0.01) breakpoints occurring at the same mat P concentrations for both the number of all taxa and high P TITAN taxa: 71, 137, 464, 627, and 1246 µg g^−1^ mat P (Table S9, Figs. 3 and 8B). WAM-mat P had a changepoint at 564 µg g^−1^ mat P, above which almost all values were high (Fig. S8). Changepoints along the mat P gradient were not observed for any other diversity, WAM, or TITAN metrics (Table S9). However, review of graphs of metrics showed a strong changepoint ≤ 500 µg g^−1^ mat P for proportion of low and high P literature-defined taxa that was particularly evident when plotted along a log mat P scale (Fig. S8d).

From the heat map (Fig. S4), changes in assemblages around 137 µg g^−1^ mat P appeared to be associated with the shift from the most likely low P co-dominants being *E. evergladianum, N. serpentiraphe,* and *Nitzschia* sp. 1 to *E. microcephala* and *B. ocalanensis*. Colonization of some high P taxa to accompany low P taxa in samples was more frequent above 137 µg g^−1^. Above 464 and especially above 627 µg g^−1^, high P taxa typically were most abundant in samples with loss of the sensitive common low P taxa *E. evergladianum, Nitzschia *sp.* 1,* and *M. calcarea*.

TITAN analysis supported the relatively high certainty of change in assemblages from low to high P taxa in the 300–600 µg g^−1^ range of mat P (Figs. 8 and S10). The peak in sum of low P taxa *z* scores was approximately 250 µg g^−1^ mat P. The spread of the interquartile range of changepoints for low P taxa, i.e., from the 0.25–0.75 cumulative frequency quartiles, was from 300–400 µg g^−1^ mat P and from 400–600 µg g^−1^ for high P taxa. The overlap in those changepoint ranges was evident in the heat map of species relative abundances in samples along the mat P gradient (Fig. S4). Between 400 and 500 µg g^−1^ mat P, as mat P increased, the frequency of high abundances of most low P taxa decreased greatly and yielded to an increase in numbers and abundances of high P taxa in samples (Fig. S4). However, some low P taxa had taxon-specific changepoints in indicator species values below the 300 µg g^−1^ mat P (Fig. S10). In contrast, some low P taxa were able to persist in relatively high mat P conditions, such as *E. microcephala* and *B. ocalanensis* that had likely indicator value changepoints greater than 500 µg g^−1^ mat P (Fig. S10). *Encyonema silesiacum* and *N. cryptotenella* had the opposite characteristics; they were high P taxa that were commonly in high abundance in low P conditions as quantified by their median indicator species thresholds near 350–400 µg g^−1^ mat P (Fig. S10).

Mat P concentrations in minimally disturbed regions of the sampling area can be used to characterize the natural variability in minimally disturbed conditions. Minimum and maximum mat P were 52 and 606 µg g^−1^ in the calibration data set at the 32 sites sampled in the FP, LM, and MN regions. The median and 75th percentile of mat P values in FP, LM, and MN were 140 and 367 µg g^−1^ mat P. Sixty-four percent of mat P values at the 32 FP, LM, and MN sites were less than 200 µg g^−1^, and 90% of mat P values were less than 500 µg g^−1^.

## Discussion

### Changes in diatom assemblages along the P gradient

Multiple lines of evidence indicated that phosphorus concentration was the determinant of changes in diatom species composition in regions of the BCNP near areas of human disturbance, as in regions of the greater Everglades. Mat P and metrics were highly related to distance from human disturbance. NMDS analyses showed mat P was the variable most strongly related to variation in diatom species composition. The mat P optima for taxa determined with the BCNP calibration dataset correlated well with P optima identified in experimental manipulations of P in the Everglades Natural Park and Everglades Water Conservation Area 2 A (Gaiser et al., 2006; Pan et al., 2000; Slate & Stevenson, 2007). Phosphorus contamination in different regions of the Everglades, typically from canal water entering marshes, is widely recognized as a major threat to biological condition in the Everglades (Gaiser et al., 2006; McCormick et al., 1996) and many other wetland ecosystems as well (Pan & Stevenson, 1996; Lougheed et al., 2007; Wyatt et al., 2010).

The number of taxa observed in BCNP samples (alpha diversity) increased with mat P concentration from an assemblage restricted to very few low P taxa to a wider diversity of taxa requiring high P concentrations, as with other regions of southern Florida (Gaiser et al., 2006; Pan et al., 2000; Raschke, 1993). Given the widespread sampling in the greater Everglades region and low number of taxa observed in low P conditions, it seems likely that diversity of diatoms is indeed low, despite limitations of 600 valve counts. Low diversity in naturally low nutrient conditions could be due to few taxa being adapted to the stress of low nutrient supply (Cardinale et al., 2009; Worm et al., 2002). Then, as nutrient concentrations increase, habitat availability increases for a larger number of taxa that require higher P to persist. Some low P taxa were observed occasionally in moderate and even high P sites; however, other low P taxa were not observed in high P sites. Although showing that taxa have indeed been extirpated from a habitat is difficult, especially for microbes for which we observe such minute proportions of populations, the lack of occurrence of taxa such as *E. evergladianum* in the large number of counts with sites having more than 550 µg g^−1^ mat P, indicates they are very rare in high P areas even though they are the most common diatom in minimally disturbed condition.

Diversity of taxa among sites (i.e., beta diversity) also increased with P concentration. Cluster analysis showed high similarity among sites occurred in low P conditions, and dissimilarity in taxonomic composition was relatively high among high P sites. This differs from other studies that show human disturbance causes greater homogenization of flora among sites (reduced beta diversity, Lougheed et al., 2008). The mechanisms regulating apparent and absolute diversity probably vary. The low P conditions in the greater Everglades that constrain taxonomic membership to a small number of taxa may be an unusually low resource condition not observed along human disturbance gradients in other locations. Thus, in the greater Everglades and BCNP we have low nutrient supply constraining taxa numbers with release of nutrient constraint as P supply increases allowing other taxa to colonize, thereby increasing apparent numbers of taxa in the habitat. In the higher range of a resource gradient that can be found in other non-Everglades locations with high human disturbance (Pan & Stevenson, 1996; Wang et al., 2006; Lougheed et al., 2007; Passy, 2008), nutrients may be sufficiently high at the high end of the human disturbance gradient so additional disturbance allows overgrowth and dominance of a few high nutrient species, thereby homogenizing biota with increasing human disturbance.

### Taxa traits and relative metric performance

More taxa were indicative of high P than low P conditions in BCNP. This is likely related to the low mat P conditions at some BCNP sites, which are among the lowest in the greater Everglades area. All taxa identified as low P and high P with TITAN and regression were characterized the same. In contrast, there were discrepancies for low and high P characterizations from the literature evaluations by NPS for BCNP when compared to the TITAN and regression traits determined with the calibration dataset. Some of the discrepancies between NPS literature and BCNP data evaluations were due to different taxonomic treatments of taxa, but many were related to the literature dealing with higher nutrient ranges, so low P (oligotrophic) taxa in the literature were from relatively low P conditions in studies which happen to be intermediate or high P conditions for BCNP and the greater Everglades. Taxa P optima were highly correlated among different Everglades projects (Gaiser et al., 2006; Hagerthey et al., 2012; Slate & Stevenson, 2007) despite the limited number of pairwise taxa comparisons across studies that were possible. The concordance of these multiple lines of evidence again indicates that phosphorus is a causal determinant of low and high P characterizations assigned to taxa.

Almost all metrics were strongly related to mat P, whether traits were derived by regression and TITAN with BCNP data or by reviewing literature. Almost all metrics with all traits differed among BCNP regions with differing levels of human disturbance. However, metrics using traits derived using regression and TITAN consistently performed better than metrics calculated with traits derived from the NPS literature evaluation. Tests with the validation dataset and the 2020 regional comparison indicated that regression and TITAN traits performed better in metrics than literature traits. As in other studies, project-specific taxonomy may affect trait assignments to species derived from the literature, but we also show environmental gradient lengths and ranges also affect trait assignment and metric performance. In comparison of regression and TITAN, TITAN traits consistently performed better.

Metrics calculated with the relative log abundance of taxa performed better than metrics calculated as the proportion of individuals or taxa. This was observed with the validation data tests of metrics relationships to mat P and differences among regions with differing mat P. Applications of simpler metrics calculated with the proportion of taxa having a trait may have good precision (low variability) when related to disturbance (Stevenson et al., 2008b) and can be easier to relate conceptually to standard characteristics of biological condition as species loss or invasion (Davies & Jackson, 2006); however, resource managers should know that the accuracy (proximity to true value) of these metrics is a concern. The actual proportions of low or high disturbance taxa could be very different if more thorough assessments than 600 valve counts were used to determine taxon presence or absence, as illustrated by the large numbers of rare taxa in log-normal, taxon-abundance distributions (Patrick, 1967; Preston, 1948).

Trait-based metrics for pollutants can be more precise indicators of pollution levels than measurements of pollutants that are difficult to measure in the environment because of spatial and temporal variability (Stevenson et al., 2010; Yuan et al., 2024). In BCNP, WAM and metrics using either TITAN or regression-derived taxa P traits were more precisely related to the human disturbance gradient (measured as latitude-longitude defined distance from the northwest corner of the study area) than measured mat P. Additionally, P concentrations in algae (mat P) or sediments are more precise and sensitive indicators of wetland P availability than measurements of P concentration in the water column (Gaiser et al., 2004; Pan et al., 2000). Thus, basing management targets on biological indicators, either as measures of biological condition or as indicators of P pollution, warrants consideration.

### Management benchmarks and metric considerations

Management benchmarks for protecting ecological condition call for determining how ecological systems respond to pollutants and relating those responses to different levels of human disturbance to prevent over- or under-protection of a resource. We were particularly interested in thresholds (tipping points, Fig. S1A-C) of ecological responses for their potential to develop consensus among stakeholders for management targets (Muradian, 2001), such as levels of pollution that protect biological condition. Many of our metrics had nonlinear relationships with untransformed mat P concentrations, either increasing or decreasing asymptotically (e.g., Fig. S1B). These responses are not as helpful for identifying threshold responses as are responses having some assimilative capacity at low levels of stressors where biota respond little because asymptotic responses are so highly sensitive to even small changes in human disturbance (Stevenson, 2011). When there is assimilative capacity for variability in physical and chemical conditions associated with human activity (Fig. S1A, C), natural variability in background conditions and small levels of human disturbance can be accommodated before tipping points are reached.

Lowess regression backed by decision tree analyses did show biological responses with assimilative capacity, such as high P taxa increasing when mat P surpassed 137 µg g^−1^ mat P and low P taxa decreasing when mat P exceeded 464 µg g^−1^. These mat P concentrations showing assimilative capacity provide breakpoints in relationships that are particularly valuable for justifying management targets in BCNP. Caution should be exercised if using decision tree analysis without looking at patterns of data in figures because decision tree can identify breakpoints at the high end (Fig. S1A, point B) or the low end of the zone of response. TITAN analysis supported these results showing transition from low to high P taxa occurring in the 300–600 µg g^−1^ mat P range with most transition of low P taxa in the 300–400 µg g^−1^ range. Cluster analysis also provided valuable benchmarks for management. Groups of sites with similar diatom assemblage composition were observed in low P conditions. For example, Group 1–1 was the low P subgroup within Group 1. The 75th percentile of mat P for Group 1–1 was 142 and for all of Group 1 (that included subgroups 1–1, 1–2, and 1–3) was 354 µg g^−1^.

Breakpoints in ecological responses of calcareous periphyton in the eastern, peat-based wetlands of the Everglades could be relevant for management of BCNP because calcareous periphyton with the same species as the Everglades is also expected in minimally disturbed conditions of BCNP. Much of the early research in the Everglades relates periphyton responses to water column TP. Floating mat cover and species composition changed greatly with elevation of TP above 10 µg L^−1^ in WCA-2A (McCormick, et al., 1996; Stevenson et al., 2002), which was important for justifying Florida’s 10 µg TP L^−1^ criterion for the Everglades. According to Hagerthey et al. (2008), mat P would be 412 µg g^−1^ in sloughs with a water column TP of 11 µg L^−1^, which was a threshold for slough macrophytes. Pan et al. (2000) found a characteristic low P periphyton assemblage (TWINSPAN I) at sites with epiphytic mat P averaging 290 µg g^−1^ ± 50 SE, which with n = 10 would have a 75th mat P percentile of 300 µg g^−1^. Gaiser et al. (2005) found enrichment of background conditions in long-term experiments by as little as 5 µg P L^−1^ can alter periphyton assemblages, and therefore, the metabolic biogeochemical processes that regulate periphyton. This sensitivity to low concentrations of P is not surprising given the asymptotic increase in periphyton growth rates and high sensitivity when P is less than 10 µg L^−1^ in stream water (Bothwell, 1989; Rier & Stevenson, 2006).

The substantial transitions from low to high P taxa in BCNP periphyton and in the Everglades occurring in the 300–400 µg g^−1^ mat P range corresponded to the 75th percentile of mat P in minimally disturbed conditions of BCNP, which was 367 µg g^−1^. Differences in periphyton in ranges of mat P less than the 75th percentile of mat P in the three most southern and eastern minimally disturbed regions of BCNP indicated that habitat specific management targets could be important. For example, if the mat P management target were set at the 367 µg g^−1^ mat P, then Group 1–1 assemblages found in very low P conditions would not be protected, even though the more inclusive Group 1 assemblages would be protected. Gaiser et al. (2006) characterized minimally disturbed conditions for Shark River Slough and Taylor Slough of the Everglades as having maximum TP at levels that would protect the Group 1–1 periphyton assemblages, so such habitats do occur on a large scale in southern Florida. Further research should be conducted to determine if lower mat P management targets should be used to protect some habitat types with the Group 1–1 periphyton assemblages. Natural habitat features could create different P conditions, which could justify setting different management targets for different kinds of wetland habitats, even though our analyses showed residuals in the metric mat P relationships with lat-long were not related to natural factors (e.g., local hydrologic conditions).

Many factors should be considered when selecting metrics for use in a monitoring program (Jackson et al., 2000). All BCNP metrics were related to mat P, differed among BCNP regions in 2020, and were related to the human disturbance gradient, in most cases. Therefore, many options could be satisfactory for use in a monitoring program. Distinguishing between metrics measuring an element of biological condition (low or high P taxa indicators) versus inferring stressors (WAM mat P models) is important conceptually relative to the idea that we want to manage stressors to protect biological condition and have clear communication with stakeholders about levels of protection for measures of biological condition (Stevenson, 2011). The higher performance of TITAN and RlogA metrics of biological condition introduces tradeoffs between using metrics that perform best (i.e., are the most accurately and precisely related to mat P concentration, changes among regions, and human disturbance) and metrics that are simpler to explain to stakeholders, i.e., how they were calculated and relate to ecological condition. Simple metrics such as numbers or even proportions of low and high P taxa may also be more vulnerable to taxonomic error, if the number of taxa distinguished among taxonomists differs. There are relatively simple ways to explain TITAN and RlogA methods and why they are likely better than other methods, but nevertheless, they are more complicated methods.

Fortunately, a library of taxa traits and a database of diatom counts allow calculation of all the metrics relatively easily, so some metrics can be used for public reports and others can be used for administrative and technical records and analysis. Such a set of tools, as we provided with this research, provides the necessary information for advancing a monitoring and assessment program and establishing management targets for biological condition and stressor levels that will protect an unimpaired condition, which is the specified goal of BCNP management (Patterson et al., 2008).

## Supplementary Information

Below is the link to the electronic supplementary material.

ESM 1 (DOCX 26.3 MB)

## Data Availability

Data are available upon request from the corresponding author.
