# Supplementary material for: Developing a monitoring and assessment program with diatoms, an improved metric calculation method, and causal analysis for Big Cypress National Preserve, Florida (USA)
Source: Environ Monit Assess. 2026 Mar 15;198(4):318. doi: 10.1007/s10661-026-15144-0 (PMC12989463; doi:10.1007/s10661-026-15144-0)
Supplement: Supplementary file 1 — (DOCX 26.3 MB) [file 10661_2026_15144_MOESM1_ESM.docx]

**Supplementary Information**

**Overview of Supplementary Information**

Our supplementary information is organized in two parts. First, we have text that describes supplementary descriptions of methods and results that were important details for developing the periphyton monitoring and assessment program. Sections of this text describe statistical analyses in more detail, metric performance for distinguishing regions (the plan for assessing condition in Big Cypress Nature Preserve, and effects of natural factors on metrics. The second part of the supplementary information provides additional figures and tables of results that are referenced in the main text of the manuscript.

**Methods**

Sampling Design

For hydrological sample years 2013 and 2014, sampling followed very similar criteria as for the entire 2013-2020 sampling period, except some sites were haphazardly selected to allow for: (a) maximizing spatial spread within a basin, (b) co-location with water-quality monitoring stations where available, (c) answering specific monitoring questions and (d) ensuring half the sites were accessible via helicopter and the other half of the sites accessible via ORV trails.

Data analysis: data selection and descriptive analyses

We constrained metric testing and analysis of thresholds in metric-mat P relationships to 3 years of data that were counted by the same taxonomist. Ordination analyses indicated taxonomist, as well as environmental factors and sampling year, were statistically related to variability in diatom species composition. Follow-up analyses of the data indicated that residuals in metric-mat P relationships also differed among sampling years and with annually varying environmental factors. Different taxonomists were assigned to count diatom samples from year to year with turnover of taxonomists present in the lab. Because interannual variation metric-mat-P relationships could not be independently assigned to either taxonomist or environment factors, we decided to constrain metric testing and threshold analysis data produced by the same taxonomist. The resulting validation dataset with 44 samples from different sites was deemed sufficient for the planned data analysis and would minimize concern about taxonomist effects.

However, we used the full calibration dataset from 2013-2020 for the preliminary descriptive analyses: ordination, cluster analysis, trait characterization, and characterization of minimally disturbed condition. Our rationale was that despite the concern about potential effect of taxonomist or interannual environmental differences, we wanted to ensure sufficient sample size to detect details in species-environment relationships and characterize traits for as many taxa as possible, particularly more rare taxa. Ongoing research is being conducted with additional years of data to investigate effects of taxonomist and interannual environmental variation on both characterizing species traits, characterizing condition at sites and in basins, and on metric performance for temporal trend analysis.

Data analysis: Metric evaluation

Because the organization of the Kruskal test comparison of metric performance methods is rather complicated, we describe them in detail. Three Kruskal tests were run to evaluate metric attributes, and each used the same set of 24 metric-mat P relationships resulting from the 24 combinations of 4 metric types, 3 trait sources, and 2 traits. To compare performance of metric types (noTaxa, PropTaxa, PropValves, RlogA) while controlling for trait source and trait, we grouped metric-mat P relationships into the 6 different trait source-trait groups (low P-Lit, low P-Regr, low P-TITAN, high P-Lit, high P-Regr, high P-TITAN). Then we ranked (1-4) the adjusted *R^2^* of the metric-mat P relationships for the 4 metric types within each of the 6 trait source-trait (TS-T) groups. In the case for the Kruskal test comparing metric types, ranks ranged from 1-4 because there were 4 metric-mat P relationships having different metric types within each TS-T group. In the Kruskal test for metric type, each metric type had 6 rankings because there were 6 TS-T groups. Using the same approach we compared performance of metrics with either Lit, Regr, or TITAN trait sources while holding metric type and trait constant; we used the Kruskal test to compare ranks (1-3 trait sources) of adjusted *R^2^* for the 3 metric-mat P relationships in the 8 metric type-trait source (MT-TS) groups (noTaxa-Lit, PropT-Lit, PropV-Lit, RLogV-Lit, noTaxa-Regr, PropT-Regr, PropV-Regr, RLogV-Regr, noTaxa-TITAN, PropT-TITAN, PropV-TITAN, RLogV-TITAN). To compare performance of metrics with either low P or high P traits while holding metric type and trait source constant, we used the Kruskal test to compare ranks (1 or 2) of adjusted *R^2^* for those 2 metric-mat P relationships in the 12 trait source– metric type groups (TS-MT groups: Lit-noTaxa, Lit-PropT, Lit-PropV, Lit-RLogV, Regr-noTaxa, Regr-PropT, Regr-PropV, Regr-RLogV, TITAN-noTaxa, TITAN-PropT, TITAN-PropV, TITAN-RLogV).

Data analysis: Metric-region relationships

Differences in metrics among regions were determined with the 2020 data to evaluate their performance and demonstrate application by determining regional differences that are related to levels of human disturbance. The regional approach was chosen because the NPS plans to analyze and report results by region using sites as replicates (a minimum of 6 sites per region (basin) each sample year), as well as to determine differences among regions and changes in time by region. Single factor ANOVA were used to compare average metric values among regions. *p* values resulting from these analyses were used to evaluate metric performance with the average *p* rank for metric types (noTaxa, PropTaxa, PropValves, and RlogA), low and high P traits, and trait sources (literature, regression, and TITAN).

Non-parametric Kruskal tests were again used, as used to compare metric-mat P relationships above, to determine which metric attributes (metric type, trait source, trait) were most important for metric performance when comparing regions. This set of three separate Kruskal tests for metric type, trait source, and trait used ranks of 24 ANOVA F-values for comparing differences in the 24 possible metrics among regions. Kruskal tests used ranks of F-values ranging from 1-4 to compare metric types within the 6 trait source-trait groups, ranging from 1-3 to compare trait sources within the 8 metric type-trait groups, and ranging from 1-2 to compare the two traits within the 12 trait source-metric type groups. In addition, we calculated an ANOVA for mat P differences among regions to compare F-values with diatom metrics to determine if diatom metrics more precisely differed among regions than mat P.

Data analysis: metric correction for natural factors

Residuals in metric-mat P relationships were related to naturally varying ecological factors to determine if expectations for metrics should be adjusted for habitat type, substrate location, substrate type, and mean water depth. Habitat type included broadleaf marsh, cypress dome, cypress scrub, graminoid marsh and mixed combinations of these habitats. Substrate locations were either floating on the water surface, on benthic sediment or soils (ground), or enveloping macrophytes (sweaters). Substrate types were filamentous macroscopic algae, microbe-dominated periphyton, and soil. Water depth was measured at the location and could covary with the major human disturbance gradient originating with human alterations in the northeast corner of the sampling area. Univariate ANOVA of residuals were used to determine separately the effects of differing habitat types, substrate locations, and substrate types on metric-mat P relationships. Linear regression in R (*lm*) was used to characterize the relationship between water depth and residuals of the metric-mat P relationship. *p* values for statistical significance were reported in results without accounting for multiple tests, but multiple tests were accounted for by dividing reported *p* values by the number of tests performed when interpreting likelihood that observed results were not due to random patterns in the data. This is like a Bonferroni correction for multiple tests. In addition, we looked at the proportion of *p* values that were less than 0.05.

Residuals in the relationships for metrics and mat P as function of latitude and longitude were related to natural environmental factors to determine if natural environmental factors affected both mat P and metrics. This second residual analysis differed from the first analysis, because it addressed whether different habitat conditions had different background P conditions and metric values versus the first analysis which evaluated whether natural environmental factors introduced bias in metric-mat P relationships.

**Results & Discussion**

Metric testing and evaluation: metric differences among regions

All metrics differed among regions with high statistical significance in 2020, with the highest P effects in the OK region and lowest in the MN region (Table S6, Fig. S6). In OK, metric values were highest for diatom inferred mat P, Shannon’s H, proportion of taxa with high P traits and relative log-transformed abundance of high P taxa valves. In OK, metric values were lowest compared to all other regions for proportion of taxa with low P traits and log-transformed valve abundances with low P traits.

Overall, OK and then EH had the highest indicators of P pollution based on relatively high richness, WAM mat P metrics, and high-P trait metrics as well as relatively low low-P metrics compared to other regions (Fig. S6). MN and LM had the consistently lowest richness, WAM mat P, and high P metrics as well as the highest low P metrics. BI, EC, and FP had intermediate levels of disturbance related to the other two groups of regions (Fig. S6).

Metric performance for detecting differences among regions was greater for the 2 diatom inferred mat P metrics (WAM_matP, WAM_RlogA.matP) compared to all other metrics as indicated by ANOVA F values (Table S6). A Kruskal test indicated ranks of metric types differed (*p*=0.025), with noTaxa and RlogA metrics having more highly ranked F values than PropTaxa and PropValves metrics (Fig. S7). Metric performance for distinguishing regions was better for TITAN and regression traits than literature traits (Fig. S7, Kruskal test, *p*=0.009). There was no difference in low and high P metrics ability to detect differences among regions (Fig. S7). Mat P was significantly different among regions (ANOVA, *p<*0.0001).

Metric testing and evaluation: corrections for natural factors

The following results describing the relationship between residuals from metric-mat P relationships and these habitat parameters should be considered with caution. Unequal sample sizes among the natural habitat features presented challenges for some residual analyses. Whereas substrate locations had from 11-21 sites for either floating, ground, or macrophyte sweaters, of the 6 habitat types there were 33 graminoid marshes and from 2-3 of all other habitat types. For substrate types, there was only one soil sample when filamentous algae and periphyton had bigger sample sizes, 7 and 36 samples, respectively. Analyzing differences between the two substrate types, filamentous algae and periphyton, was an issue because shifts from microbial periphyton to filamentous macroalgae are associated with increasing P concentrations. Similarly, in this dataset water depth can be associated with human disturbance.

Little evidence indicated that natural factors affected metric-mat P relationships (Tables S7, S8). Water depth was not significantly (*p*<0.05) related to residuals in any metric-mat P relationship (Table S7). Time of year sampled (indicated by the variable named season) had a significant (*p*<0.05) negative effect on 6 of 29 metrics, which were metrics for the numbers of high P taxa and diversity (Table S7). For substrate type, location, and hydrologic year, *p* values for statistical significance were seldom less than 0.05 (1-4 of 29 metrics, respectively) and not less than 0.05 if accounting for the multiple statistical tests conducted (Table S8). For habitat type, 5 of 29 metrics had a *p* value less than 0.05, but low sample size for habitats other than graminoid marshes limited certainty in this observation.

Evidence was weak for natural factors affecting metrics after the effects of human disturbance (lat-long model) were accounted for. Residuals from relationships relating either metrics or mat P with the lat-long model were so seldom related to natural factors with ANOVA or regression that the few tests with p<0.05 could have occurred by chance. Attained significance (*p*) was not greater than 0.05 for any of the mat P or metric residuals relationships with water depth, time of season when sampling occurred, or substrate type. *p* was < 0.05 for 1, 5, and 8 of the residual relationships with habitat type, substrate location, and hydrologic year, respectively (Table S8). Thus, substrate location and hydrologic year were the most likely natural factors affecting mat P and metrics. Interestingly, lat-long related residuals for both WAM metrics and mat P were related to hydrologic year, but residuals for regression or TITAN metrics were not related to hydrologic year (Table S8).

Therefore, metrics were affected little by natural factors such as wetland habitat type, substrate type, substrate location, water depth, and when sampling occurred during the high-water season. Analyses of both sets of residuals, for metric-mat P relationships or metric and mat P relationships with the lat-long models of human disturbance, showed little likelihood that natural factors had major effects on metrics. Metrics are often robust to pollution levels and human disturbance, but there are large-scale studies when we need to account for how minimally disturbed condition of pollutants and metrics vary with natural factors, such as in streams and lakes assessments (Cao et al., 2007; Stevenson et al., 2013; Tang et al., 2016). But the range of natural factors in BCNP had little effect on metric relationships to mat P (the stressor) or the lat-long model of human disturbance.

Hydrologic year did affect expected levels of mat P and metrics in BCNP according to differences in residuals for the metric and mat P relationships with the lat-long model. All years of samples were counted by the same taxonomist for data used in metric testing and evaluation, which reduces the likelihood that taxonomist caused interannual variability in residuals. Interannual differences in P availability seem to be the issue because mat P residuals also differed with hydrologic year. Perhaps weather-related events, such as long periods of stable weather or recent rainfalls, affected P availability and thereby, metric values. Long periods of stable weather with low external P loadings could allow biological uptake to reduce P in the water column. Rainfall and accompanying runoff could increase P in the water column.

**References for above**

Cao, Y., Hawkins, C. P., Olson, J., & Kosterman, M. A. (2007). Modeling natural environmental gradients improves the accuracy and precision of diatom-based indicators. *Journal of the North American Benthological Society, 26*(3), 566-585.

Stevenson, R. J., Zalack, J., & Wolin, J. (2013). A multimetric index of lake diatom condition using surface sediment assemblages. *Freshwater Science, 32*, 1005–1025.

Tang, T., Stevenson, R. J., & Infante, D. M. (2016). Accounting for regional variation in both natural environment and human disturbance to improve performance of multimetric indices of lotic benthic diatoms. *Science of the Total Environment 268*, 1124-1134.

**Supplementary Figures and Tables**

Table S1. Literature citations used to determine NPS literature trait characterizations. The number and identify of the species found in references is listed. Full references follow this table.

| **Author** | **Year** | **N Spp** | **Species** |
| --- | --- | --- | --- |
| Swift, D. R., and R. B. Nicholas. | 1987 | 1 | *Diadesmis confervacea* |
| Whitmore, T.J. | 1989 | 21 | *Achnanthidium minutissimum, Amphora ovalis, Asterionella ralfsii, Cyclotella meneghiniana, Denticula elegans, Eunotia formica, Eunotia glacialis, Eunotia incisa, Eunotia monodon, Eunotia naegelii, Eunotia pectinalis, Eunotia pectinalis var. undulata, Fragilaria crotonensis, Fragilaria vaucheriae, Hantzschia amphioxys, Melosira varians, Sellaphora pupula var. rectangularis, Synedra acus, Synedra delicatissima var. angustissima, Synedra rumpens, Synedra ulna* |
| Raschke, R. L. | 1993 | 2 | *Eunotia flexuosa, Navicula cryptotenella* |
| Van Dam, H., A. Mertens, and J. Sinkeldam | 1994 | 17 | *Achnanthes coarctata, Achnanthes minutissima var. jackii, Achnanthidium caledonicum, Achnanthidium exiguum, Achnanthidium exilis, Achnanthidium minutissimum, Adlafia bryophila, Adlafia minuscula, Caloneis bacillum, Cocconeis placentula var. lineata, Craticula cuspidata, Craticula molestiformis, Cymbella aspera, Cymbella delicatula, Cymbella gracilis, Cymbella mesiana, Cymbella proxima, Diploneis oblongella, Gomphonema minutum, Nitzschia acidoclinata* |
| McCormick, P. V., and R .J. Stevenson | 1998 | 2 | *Nitzschia filiformis, Sellaphora minima* |
| Cooper, S. R., J. Huvane, P. Vaithiyanathan, and C. J. Richardson | 1999 | 41 | *Achnanthidium caledonicum, Achnanthidium exiguum, Adlafia minuscula, Amphora veneta, Anomoeoneis sphaerophora f. costata, Caloneis bacillum, Cocconeis placentula var. lineata, Cyclotella meneghiniana, Cymbella mesiana, Denticula kuetzingii, Diadesmis confervacea, Diploneis elliptica, Encyonema evergladianum, Encyonopsis microcephala, Eunotia camelus, Eunotia glacialis, Eunotia naegelii, Fragilaria capucina var. gracilis, Fragilaria capucina var. mesolepta, Fragilaria synegrotesca, Gomphonema gracile, Gomphonema parvulum, Hantzschia amphioxys, Kobayasiella subtilissima, Mastogloia smithii, Navicula cryptocephala, Nitzschia amphibia, Nitzschia frustulum, Nitzschia intermedia, Nitzschia linearis, Nitzschia nana, Nitzschia palea, Pinnularia divergens, Pinnularia gibba, Pinnularia viridis, Rhopalodia gibba, Sellaphora minima, Sellaphora pupula, Sellaphora seminulum, Synedra ulna* |
| Pan, Y., R. J. Stevenson, P. Vaithiyanathan, J. Slate, and C. J. Richardson | 2000 | 2 | *Gomphonema gracile, Nitzschia amphibia f. frauenfeldii* |
| Slate, J. E., and R. J. Stevenson | 2000 | 11 | *Caloneis bacillum, Cocconeis placentula var. lineata, Craticula cuspidata, Cyclotella meneghiniana, Epthemia adnata, Eunotia formica, Lemnicola hungarica, Navicula cryptocephala, Nitzschia frustulum, Sellaphora minima, Synedra ulna* |
| Winter, J. G., and H. C. Duthie | 2000 | 2 | *Achnanthidium minutissimum, Amphora pediculus* |
| Gaiser, E. E., J. C. Trexler, R. D. Jones, D. L. Childers, J. H. Richards, and L. J. Scinto | 2006 | 12 | *Achnanthidium caledonicum, Amphora veneta, Encyonema evergladianum, Encyonopsis microcephala, Eunotia flexuosa, Eunotia incisa, Fragilaria synegrotesca, Gomphonema parvulum, Mastogloia smithii, Navicula cryptotenella, Nitzschia amphibia, Rhopalodia gibba* |
| Potapova, M., and D. F. Charles | 2007 | 125 | *Achnanthes minutissima var. jackii, Achnanthidium caledonicum, Achnanthidium exiguum, Achnanthidium exilis, Achnanthidium minutissimum, Adlafia bryophila, Adlafia minuscula, Amphora copulata, Amphora ovalis, Amphora pediculus, Amphora veneta, Bacillaria paradoxa, Brachysira microcephala, Caloneis bacillum, Cocconeis placentula var. lineata, Craticula accomoda, Craticula cuspidata, Craticula molestiformis, Cymbella delicatula, Cymbella mesiana, Denticula elegans, Denticula kuetzingii, Diadesmis confervacea, Diploneis oblongella, Diploneis parma, Encyonema auerswaldii, Encyonema evergladianum, Encyonema minutum, Encyonema silesiacum, Encyonopsis cesatii, Encyonopsis microcephala, Epthemia adnata, Epithemia sorex, Eunotia bilunaris, Eunotia flexuosa, Eunotia incisa, Eunotia monodon, Eunotia pectinalis, Eunotia prarerupta, Fragilaria capucina var. gracilis, Fragilaria crotonensis, Fragilaria synegrotesca, Fragilaria tenera, Fragilaria vaucheriae, Gomphonema affine, Gomphonema angustatum, Gomphonema calvatum, Gomphonema gracile, Gomphonema insigne, Gomphonema intricatum, Gomphonema mexicanum, Gomphonema minutum, Gomphonema olivaceoides, Gomphonema parvulum, Gomphonema pumilum, Gomphoenma sarcophagus, Gomphonema truncatum, Gomphosphenia grovei, Gyrosigma acuminatum, Hantzschia amphioxys, Hippodonta capitata, Hippodonta hungarica, Luticola mutica, Luticola naviculoides, Mastogloia smithii, Melosira varians, Navicula arvensis f. major, Navicula cari, Navicula cincta, Navicula cryptocephala, Navicula cryptotenella, Navicula erifuga, Navicula exilis, Navicula germainii, Navicula gregaria, Navicula lanceolata, Navicula laterostrata, Navicula menisculus, Navicula notha, Navicula radiosa, Navicula recens, Naviucla rostellata, Navicula stroemii, Navicula submuralis, Navicula trivialis, Navicula wallacei, Neidium dubium, Nitzschia acicularis, Nitzschia amphibia, Nitzschia amphibia f. frauenfeldii, Nitzschia angustata, Nitzschia archibaldii, Nitzschia capitellata, Nitzschia filiformis, Nitzschia fonticola, Nitzschia frustulum, Nitzschia hantzschiana, Nitzschia incognita, Nitzschia intermedia, Nitzschia lacuum, Nitzschia liebethruthii, Nitzschia linearis, Nitzschia palea, Nitzschia palea var. debilis, Nitzschia perminuta, Nitzschia sigmoidea, Nitzschia supralitorea, Nitzschia tropica, Nitzschia vitrea, Pinnularia microstauron, Placoneis exigua, Planothidium rostratum, Psammothidium marginulatum, Pseudostaurosira brevistriata, Rhoicosphenia abbreviata, Rhopalodia brebissonii, Rhopalodia gibba, Sellaphora bacillum, Sellaphora laevissima, Sellaphora pupula, Sellaphora pupula var. rectangularis, Sellaphora seminulum, Synedra acus, Synedra delicatissima var. angustissima, Synedra ulna* |
| Slate, J. E., and R. J. Stevenson | 2007 | 49 | *Achnanthidium caledonicum, Achnanthidium exiguum, Amphora veneta, Caloneis bacillum, Cocconeis placentula var. lineata, Craticula cuspidata, Craticula molestiformis, Cyclotella meneghiniana, Diadesmis confervacea, Diploneis oblongella, Diploneis ovalis, Encyonema neomesianum, Encyonopsis microcephala, Epithemia spp., Eunotia camelus, Eunotia flexuosa, Eunotia formica, Eunotia glacialis, Fragilaria capucina var. gracilis, Fragilaria synegrotesca, Fragilaria tenera, Gomphonema affine, Gomphonema gracile, Gomphonema parvulum, Gomphonema tenellum, Gomphonema vibriodes, Hantzschia amphioxys, Kobayasiella subtilissima, Lemnicola hungarica, Mastogloia smithii, Navicula cryptocephala, Navicula cryptotenella, Navicula podzorskii, Nitzschia amphibia f. frauenfeldii, Nitzschia frustulum, Nitzschia nana, Nitzschia palea var. debilis, Nitzschia perminuta, Nitzschia radicula, Nitzschia serpentiraphe, Nupela silvahercynia, Pinnularia acrosphaeria, Pinnularia divergens, Rhopalodia gibba, Sellaphora laevissima, Sellaphora minima, Sellaphora seminulum, Synedra acus, Synedra ulna* |
| Zalack, J., and R. J. Stevenson | 2010 | 1 | *Mastogloia smithii* |

**References for Table S1**

Cooper, S. R., J. Huvane, P. Vaithiyanathan, and C. J. Richardson. 1999. Calibration of diatos along a nutrient gradient in Florida Everglades Water Conservation Area-2A, USA. Journal of Paleolimnology 22:413-437.

Gaiser, E. E., D. L. Childers, R. D. Jones, J. H. Richards, and L. J. Scinto. 2006. Periphyton responses to eutrophication in the Florida Everglades: cross-system patterns of strucural and composition changes. Limnology & Oceanography 51:617-630.

McCormick, P. V., and R. J. Stevenson. 1998. Periphyton as a tool for ecological assessment and management in the Florida Everglades. Journal of Phycology 34:726-733.

Pan, Y., R. J. Stevenson, P. Vaithiyanathan, J. Slate, and C. J. Richardson. 1997. Using experimental and observational approaches to determine causes of algal changes in the Everglades. in C. J. Richardson (editor). 1996-1997 Biennial Report: Effects of phosphorus and hydroperioda alterations on ecosystem structure and function in the Everglades. Duke Wetland Center Publication 97-05, Durham, North Carolina.

Potapova, M., and D. F. Charles. 2007. Diatom metrics for monitoring eutrophication in rivers of the United States. Ecological Indicators 7:48-70.

Raschke, R. L. 1993. Diatom (Bacillariophyta) community response to phosphorus in the Everglades National Park USA. Phycologia 32:48-58.

Slate, J., and R. J. Stevenson. 2000. Recent and abrupt environmental change in the Florida Everglades indicated from siliceous microfossils. Wetlands 20:346-356.

Slate, J. E., and R. J. Stevenson. 2007. The diatom flora of phosphorus-enriched and unenriched sites in an Everglades marsh. Diatom Research 22:355-386.

Swift, D. R., and R. B. Nicholas 1987. Periphyton and Water Quality Relationships in the Everglades Water Conservation Areas 1978-1982. Technical Publication. South Florida Water Management District.

Van Dam, H., A. Mertenes, and J. Sinkeldam. 1994. A coded checklist and ecological indicator values of freshwater diatoms from the Netherlands. Netherlands Journal of Aquatic Ecology 28:117-33.

Whitmore, T. J. 1989. Flordia diatom assemblages as indicators of trophic state and pH. Limnology & Oceanography 34(5):882-95.

Winter, J. G., and H. C. Duthie. 2000. Stream epilithic, epipelic, and epiphytic diatoms: habitat fidelity and use in biomonitoring. Aquatic Ecology 34:345-53.

Zalack, J. and R.J. Stevenson. 2010. Status of Periphyton Assemblages in Big Cypress National Preserve: A preliminary examination of periphyton communities in domes and rairies along an impact gradient. Report from Michigan State University to the National Park Service.


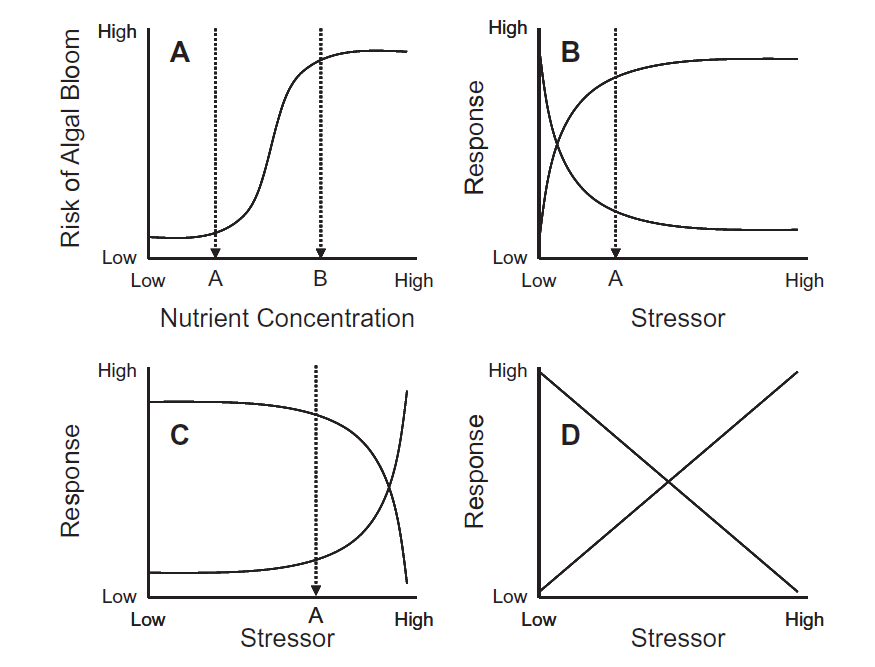


Fig. S1. Figures indicating the range of stressor–response relationships possible from Stevenson (2011). Figures are drawn to illustrate changes in algal bloom risk with increasing nutrient concentrations, but they could apply to any set of dependent and independent variables. The vertical, dotted arrows indicate thresholds along the stressor gradient where great changes in a response occur, either as stressors increase with greater contamination or decrease with restoration successfully reducing the stressor.


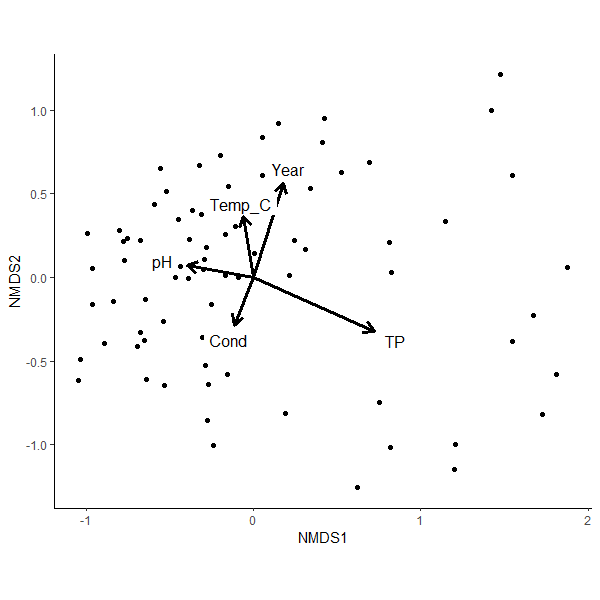


Fig. S2. Graphical results of NMDS ordination of 78 samples with all chemistries from the calibration dataset.

Table S2. Strength of ecological factor and taxonomist relationships with NMDS axes indicated by the coefficient of determination (*R^2^*) and associated attained significance (*p*).

| Variable | *R^2^* | *p* |
| --- | --- | --- |
| mat P | 0.624 | 0.001 |
| Temp_C | 0.128 | 0.005 |
| pH | 0.162 | 0.002 |
| Conductivity | 0.086 | 0.041 |
| H20 Year | 0.349 | 0.001 |
| H2O_Pres | 0.000 | 1.000 |
| Habitat | 0.273 | 0.001 |
| Color | 0.161 | 0.001 |
| taxonomist | 0.173 | 0.001 |


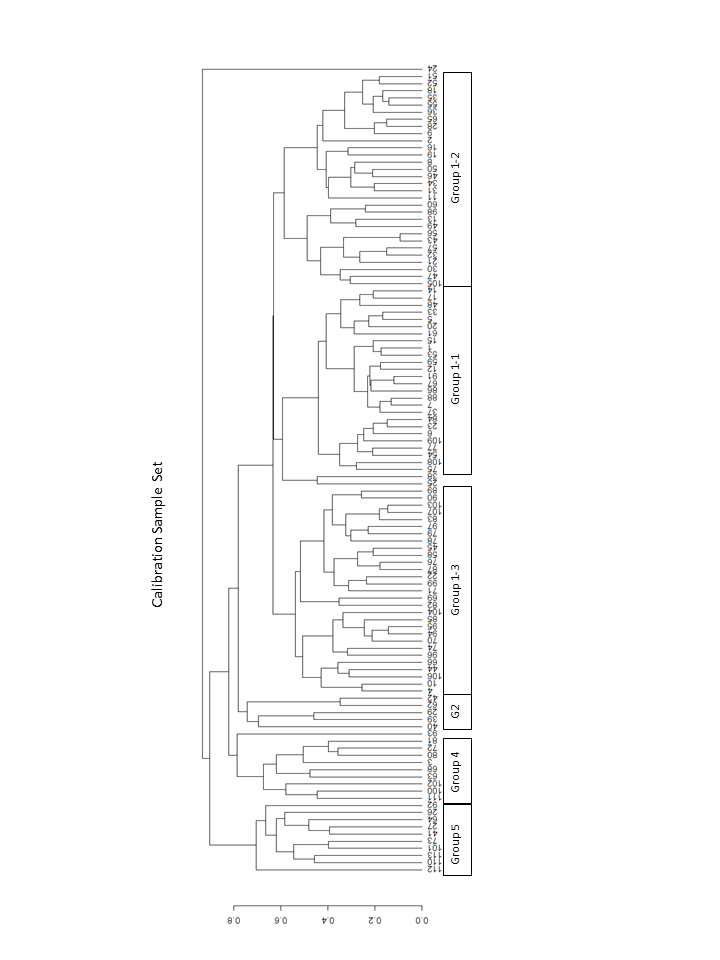


Fig. S3. Results of cluster analysis with Bray Curtis formula and all 113 samples from different sites in the calibration data set.


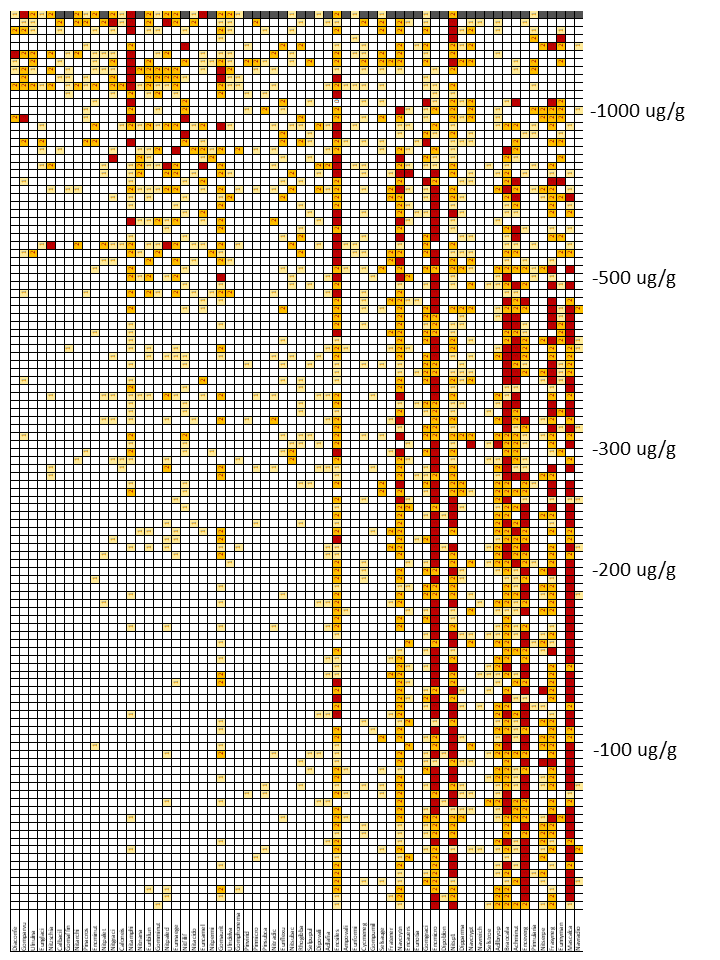


Fig. S4. a) Heat map of species relative abundances in calibration dataset along mat P gradient. Proportions of counts: 0 if white; >0 & ≤0.01 if yellow; >1 & ≤ 10 if golden; >10% if red.

Fig. S4. b) Species names associated with codes and rows of relative abundances in Fig. S4a. Species names for rows in Fig. S4 can be matched here using the color patterns of cells in the first 4 and last 4 samples that are shown here in the heat map (Fig. S4a).

Table S3. Low and high P taxa traits determined by literature (Lit), regression (Regr), and TITAN, as well as taxa mat P optima. Taxa are listed in order of abundance determined by the sum of taxon relative abundances in all calibration data set samples for the first 24 taxa, then alphabetically if they were observed in the dataset, and then alphabetically if they were not observed in the dataset by had traits determined by the literature. 1 indicates either a low or high P trait is present and 0 indicates it is absent. The number of samples in which a taxon was observed is indicated by NoSamp. RAsum is the sum of taxa relative abundances in all samples from 2013-2020. Given 238 samples in the full 2013-2020 dataset, dividing RAsum by 238 gives the average proportional relative abundance for taxa. Dividing RAsum by NoSamp gives the average proportional relative abundance of taxa in samples in which it was observed.

| Harmonized_Name | NoSamp | RAsum | Low P | | | High P | | | Optima |
| --- | --- | --- | --- | --- | --- | --- | --- | --- | --- |
|  |  |  | Lit | Regr | TITAN | Lit | Regr | TITAN |  |
| Encyonopsis microcephala (Grunow) Krammer | 210 | 44.433 | 1 | 1 | 1 | 0 | 0 | 0 | 7.787 |
| Mastogloia calcarea Lee, Gaiser, Van de Vijver, Edlund, et Spauld. | 176 | 34.542 | 1 | 1 | 1 | 0 | 0 | 0 | 7.320 |
| Encyonema evergladianum Krammer | 157 | 22.873 | 1 | 1 | 1 | 0 | 0 | 0 | 6.818 |
| Brachysira ocalanensis Shayler and Siver | 206 | 17.440 |  | 1 | 1 |  | 0 | 0 | 7.723 |
| Nitzschia sp. 1 | 218 | 17.251 |  | 1 | 1 |  | 0 | 0 | 7.610 |
| Fragilaria synegrotesca Lange-Bertalot | 175 | 10.436 | 1 | 1 | 1 | 0 | 0 | 0 | 8.074 |
| Adlafia bryophila (Petersen) Moser, Lange-Bertalot and Metzeltin | 144 | 3.831 |  | 1 | 1 |  | 0 | 0 | 7.510 |
| Nitzschia serpentiraphe Lange-Bertalot | 69 | 2.184 |  | 1 | 1 |  | 0 | 0 | 7.245 |
| Sellaphora stroemii (Hustedt) Kobayasi | 27 | 0.179 |  |  | 1 |  |  | 0 | 7.246 |
| Diploneis oblongella (Nägeli ex Kützing) Ross | 13 | 0.047 | 1 |  | 1 | 0 |  | 0 | 6.794 |
| Navicula reichardtiana Lange-Bertalot | 10 | 0.034 |  |  | 1 |  |  | 0 | 7.728 |
| Encyonema silesiacum (Bleisch) Mann | 224 | 16.305 | 1 | 0 | 0 | 0 | 1 | 1 | 8.780 |
| Navicula cryptotenella Lange-Bertalot | 211 | 12.763 |  | 0 | 0 |  | 1 | 1 | 8.484 |
| Achnanthidium minutissimum (Kützing) Czarnecki | 170 | 11.607 |  |  | 0 |  |  | 1 | 8.435 |
| Nitzschia amphibia Grunow | 109 | 7.513 | 0 | 0 | 0 | 1 | 1 | 1 | 10.227 |
| Gomphonema auritum Braun | 133 | 3.546 |  | 0 | 0 |  | 1 | 1 | 9.374 |
| Gomphonema gracile Ehrenberg | 133 | 2.950 |  | 0 | 0 |  | 1 | 1 | 8.776 |
| Eunotia nymanniana Grunow | 48 | 2.565 |  | 0 | 0 |  | 1 | 1 | 10.166 |
| Nitzschia palea var. debilis (Kützing) Grunow | 67 | 2.418 | 0 | 0 | 0 | 1 | 1 | 1 | 9.680 |
| Eunotia naegelii Migula | 76 | 2.030 | 0 | 0 | 0 | 1 | 1 | 1 | 9.669 |
| Eunotia camelus Ehrenberg | 37 | 1.637 |  | 0 | 0 |  | 1 | 1 | 9.401 |
| Nitzschia filiformis (W. Smith) Schütt | 37 | 1.622 | 0 | 0 | 0 | 1 | 1 | 1 | 10.214 |
| Gomphonema parvulum (Kützing) Kützing | 33 | 1.574 | 0 | 0 | 0 | 1 | 1 | 1 | 10.516 |
| Navicula cryptocephala Kützing | 57 | 1.220 |  |  | 0 |  |  | 1 | 8.966 |
| Achnanthidium | 12 | 0.101 |  | 0 |  |  | 1 |  | 10.803 |
| Achnanthidium exiguum (Grunow) Czarnecki | 3 | 0.020 | 0 |  |  | 1 |  |  | 8.381 |
| Achnanthidium rosenstockii | 1 | 0.003 |  |  |  |  |  |  |  |
| Adlafia | 47 | 0.300 |  |  |  |  |  |  | 8.727 |
| Amphora | 1 | 0.002 |  |  |  |  |  |  | 8.614 |
| Amphora copulata (Kützing) Schoeman and Archibald | 6 | 0.022 | 0 |  |  | 1 |  |  | 7.525 |
| Amphora ovalis (Kützing) Kützing | 10 | 0.038 |  |  |  |  |  |  | 8.640 |
| Anomoeoneis | 1 | 0.002 |  |  |  |  |  |  |  |
| Aulacoseira | 4 | 0.437 |  |  |  |  |  |  | 10.780 |
| Aulacoseira ambigua (Grunow) Simonsen | 1 | 0.007 |  |  |  |  |  |  | 9.937 |
| Aulacoseira granulata (Ehrenberg) Simonsen | 1 | 0.003 |  |  |  |  |  |  |  |
| Aulacoseira italica (Ehrenberg) Simonsen | 1 | 0.005 |  |  |  |  |  |  | 11.764 |
| Brachysira | 4 | 0.008 |  |  |  |  |  |  |  |
| Brachysira vitrea (Grunow) Ross | 3 | 0.021 |  |  |  |  |  |  |  |
| Caloneis | 11 | 0.059 |  | 0 | 0 |  | 1 | 1 | 10.071 |
| Caloneis alpestris (Grunow) Cleve | 1 | 0.025 |  |  |  |  |  |  | 9.647 |
| Caloneis bacillum (Grunow) Cleve | 12 | 0.122 | 0 | 0 |  | 1 | 1 |  | 11.384 |
| Caloneis fontinalis (Grunow) Cleve-Euler | 2 | 0.048 |  |  |  |  |  |  | 10.022 |
| Chamaepinnularia | 3 | 0.010 |  |  |  |  |  |  | 7.021 |
| Chamaepinnularia mediocris (Krasske) Lange-Bertalot | 4 | 0.036 |  |  |  |  |  |  | 9.175 |
| Chamaepinnularia submuscicola (Krasske) Lange-Bertalot | 3 | 0.009 |  |  |  |  |  |  | 8.373 |
| Cocconeis placentula Ehrenberg | 4 | 0.007 |  |  |  |  |  |  | 8.812 |
| Craticula cuspidata (Kützing) Mann | 4 | 0.014 | 0 |  |  | 1 |  |  | 8.797 |
| Craticula halophila (Grunow ex Van Heurck) Mann | 1 | 0.002 |  |  |  |  |  |  | 8.012 |
| Craticula subminuscula (Manguin) Wetzel et al. | 1 | 0.006 |  |  |  |  |  |  |  |
| Cyclotella meneghiniana Kützing | 76 | 0.368 | 0 | 0 | 0 | 1 | 1 | 1 | 9.524 |
| Cymbella | 3 | 0.012 |  |  |  |  |  |  | 10.284 |
| Cymbella aspera (Ehrenberg) Cleve | 2 | 0.006 |  |  |  |  |  |  | 9.260 |
| Cymbella leptoceros (Ehrenberg) Kützing | 4 | 0.028 |  |  |  |  |  |  | 8.814 |
| Cymbella mexicana (Ehrenberg) Cleve | 2 | 0.010 |  |  |  |  |  |  | 9.525 |
| Diadesmis confervacea Kützing | 10 | 0.705 | 0 | 0 | 0 | 1 | 1 | 1 | 11.057 |
| Diploneis | 2 | 0.003 |  |  |  |  |  |  | 6.566 |
| Diploneis elliptica (Kützing) Cleve | 4 | 0.008 |  |  |  |  |  |  | 7.045 |
| Diploneis marginestriata Hustedt | 1 | 0.003 |  |  |  |  |  |  | 9.402 |
| Diploneis ovalis (Hilse) Cleve | 51 | 0.218 |  |  | 0 |  |  | 1 | 8.726 |
| Diploneis parma Cleve | 46 | 0.349 | 1 |  |  | 0 |  |  | 8.424 |
| Diploneis puella (Schumann) Cleve | 1 | 0.012 |  |  |  |  |  |  | 9.647 |
| Diploneis subovalis Cleve | 1 | 0.003 |  |  |  |  |  |  | 6.634 |
| Encyonema | 5 | 0.023 | 1 |  |  | 0 |  |  | 7.473 |
| Encyonema auerswaldii Rabenhorst | 61 | 0.665 |  |  | 0 |  |  | 1 | 8.337 |
| Encyonema cespitosum | 1 | 0.002 |  |  |  |  |  |  | 5.922 |
| Encyonema minutum (Hilse) Mann | 25 | 0.264 | 1 | 0 | 0 | 0 | 1 | 1 | 10.056 |
| Encyonema pergracile Krammer | 1 | 0.002 |  |  |  |  |  |  |  |
| Encyonopsis | 2 | 0.006 |  |  |  |  |  |  | 6.357 |
| Encyonopsis cesatii (Rabenhorst) Krammer | 4 | 0.009 | 1 |  |  | 0 |  |  | 9.243 |
| Epithemia | 2 | 0.006 |  |  |  |  |  |  |  |
| Epithemia adnata (Kützing) Brébisson | 1 | 0.015 |  |  |  |  |  |  | 9.937 |
| Eunotia | 27 | 0.095 |  | 0 | 0 |  | 1 | 1 | 9.517 |
| Eunotia arcus Ehrenberg | 7 | 0.029 |  |  |  |  |  |  | 9.175 |
| Eunotia bilunaris (Ehrenberg) Souza | 61 | 0.842 |  | 0 | 0 |  | 1 | 1 | 9.981 |
| Eunotia flexuosa (Brébisson ex Kutzing) Kützing | 26 | 0.397 |  | 0 | 0 |  | 1 | 1 | 9.706 |
| Eunotia formica Ehrenberg | 10 | 0.049 | 0 | 0 | 0 | 1 | 1 | 1 | 10.366 |
| Eunotia glacialis Meister | 14 | 0.074 |  |  | 0 |  |  | 1 | 9.711 |
| Eunotia implicata Nörpel, Alles and Lange-Bertalot | 2 | 0.006 |  |  |  |  |  |  |  |
| Eunotia intermedia (Krasske ex Hustedt) Nörpel and Lange-Bertalot | 3 | 0.014 |  |  |  |  |  |  |  |
| Eunotia minor (Kützing) Grunow | 5 | 0.025 |  |  |  |  |  |  | 6.268 |
| Eunotia monodon Ehrenberg | 3 | 0.022 | 1 |  |  | 0 |  |  | 9.825 |
| Eunotia parallela Ehrenberg | 1 | 0.013 |  |  |  |  |  |  | 8.614 |
| Eunotia pectinalis (Kützing) Rabenhorst | 2 | 0.008 | 0 |  |  | 1 |  |  |  |
| Eunotia pectinalis var. undulata (Ralfs) Rabenhorst | 3 | 0.005 | 0 |  |  | 1 |  |  | 9.012 |
| Eunotia pirla Carter and Flower | 2 | 0.013 |  |  |  |  |  |  |  |
| Eunotia rhomboidea Hustedt | 3 | 0.006 |  |  |  |  |  |  | 8.809 |
| Eunotia subarcuatoides Alles, Nörpel and Lange-Bertalot | 7 | 0.064 |  |  |  |  |  |  | 9.961 |
| Fallacia lenzii (Hustedt) Lange-Bertalot | 3 | 0.005 |  |  |  |  |  |  | 7.813 |
| Fragilaria | 13 | 0.151 |  |  |  |  |  |  | 7.424 |
| Fragilaria tenera (Smith) Lange-Bertalot | 47 | 0.848 | 1 |  |  | 0 |  |  | 8.799 |
| Fragilaria vaucheriae (Kützing) Petersen | 3 | 0.008 |  |  |  |  |  |  | 8.773 |
| Frustulia | 1 | 0.005 |  |  |  |  |  |  | 9.625 |
| Frustulia crassinervia (Brébisson) Lange-Bertalot and Krammer | 1 | 0.013 |  |  |  |  |  |  |  |
| Gomphonema | 25 | 0.149 |  |  | 0 |  |  | 1 | 9.264 |
| Gomphonema acuminatum Ehrenberg | 5 | 0.079 |  |  |  |  |  |  | 7.887 |
| Gomphonema affine Kützing | 21 | 0.266 |  | 0 | 0 |  | 1 | 1 | 10.272 |
| Gomphonema contraturris Lange-Bertalot and Reichardt | 11 | 0.065 |  | 0 |  |  | 1 |  | 10.539 |
| Gomphonema drutelingense Reichardt | 6 | 0.016 |  |  |  |  |  |  | 10.136 |
| Gomphonema intricatum Kützing | 1 | 0.002 |  |  |  |  |  |  |  |
| Gomphonema minutum (Agardh) Agardh | 39 | 0.503 | 0 | 0 | 0 | 1 | 1 | 1 | 9.960 |
| Gomphonema pumilum (Grunow) Reichardt and Lange-Bertalot | 10 | 0.043 | 1 |  |  | 0 |  |  | 8.082 |
| Gomphonema subclavatum (Grunow) Grunow | 2 | 0.004 |  |  |  |  |  |  | 12.027 |
| Gomphonema truncatum Ehrenberg | 1 | 0.002 |  |  |  |  |  |  | 9.647 |
| Gyrosigma acuminatum (Kützing) Rabenhorst | 1 | 0.003 |  |  |  |  |  |  |  |
| Halamphora veneta (Kützing) Levkov | 4 | 0.047 |  |  |  |  |  |  | 10.850 |
| Hantzschia amphioxys (Ehrenberg) Grunow | 1 | 0.002 | 0 |  |  | 1 |  |  | 10.353 |
| Hippodonta capitata (Ehrenberg) Lange-Bertalot, Metzeltin and Witkowski | 1 | 0.003 |  |  |  |  |  |  |  |
| Hippodonta lueneburgensis (Grunow) Lange-Bertalot, Metzeltin and Witkowski | 1 | 0.014 |  |  |  |  |  |  | 9.066 |
| Humidophila perpusilla (Grunow) Lowe et al. | 1 | 0.023 |  |  |  |  |  |  | 7.856 |
| Lemnicola hungarica (Grunow) Round and Basson | 1 | 0.003 |  |  |  |  |  |  |  |
| Luticola | 2 | 0.004 |  |  |  |  |  |  | 9.964 |
| Luticola naviculoides Johansen | 1 | 0.060 | 1 |  |  | 0 |  |  |  |
| Navicula | 8 | 0.110 |  |  |  |  |  |  | 8.462 |
| Navicula aquaeductae (Krasske) Krasske | 1 | 0.002 |  |  |  |  |  |  | 7.856 |
| Navicula capitatoradiata Germain | 1 | 0.002 |  |  |  |  |  |  | 9.937 |
| Navicula caterva Hohn and Hellerman | 1 | 0.003 |  |  |  |  |  |  |  |
| Navicula contenta var. biceps (Grunow) Van Heurck | 1 | 0.003 |  |  |  |  |  |  |  |
| Navicula exilis Kützing | 2 | 0.004 |  |  |  |  |  |  | 9.570 |
| Navicula lanceolata (Agardh) Kützing | 3 | 0.014 | 0 |  |  | 1 |  |  |  |
| Navicula oligotraphenta Lange-Bertalot and Hofmann | 2 | 0.006 |  |  |  |  |  |  | 7.603 |
| Navicula radiosa Kützing | 20 | 0.119 |  |  |  |  |  |  | 7.740 |
| Navicula recens (Lange-Bertalot) Lange-Bertalot | 5 | 0.033 | 0 |  |  | 1 |  |  | 8.592 |
| Navicula trivialis Lange-Bertalot | 4 | 0.013 | 0 |  |  | 1 |  |  | 8.408 |
| Navicula veneta Kützing | 4 | 0.043 |  |  |  |  |  |  | 8.835 |
| Neidium | 1 | 0.008 |  |  |  |  |  |  | 9.066 |
| Neidium affine (Ehrenberg) Pfitzer | 2 | 0.003 |  |  |  |  |  |  |  |
| Neidium ampliatum (Ehrenberg) Krammer | 3 | 0.005 |  |  |  |  |  |  | 9.022 |
| Neidium iridis (Ehrenberg) Cleve | 1 | 0.007 |  |  |  |  |  |  | 10.627 |
| Nitzschia | 32 | 0.876 |  |  | 0 |  |  | 1 | 9.554 |
| Nitzschia acicularis (Kützing) Smith | 5 | 0.026 | 0 | 0 |  | 1 | 1 |  | 11.871 |
| Nitzschia acidoclinata Lange-Bertalot | 26 | 0.725 |  |  | 0 |  |  | 1 | 9.801 |
| Nitzschia agnita Hustedt | 6 | 0.034 |  | 0 |  |  | 1 |  | 10.556 |
| Nitzschia archibaldii Lange-Bertalot | 21 | 0.539 | 0 | 0 | 0 | 1 | 1 | 1 | 10.367 |
| Nitzschia brevissima Grunow | 1 | 0.003 |  |  |  |  |  |  |  |
| Nitzschia capitellata Hustedt | 1 | 0.003 | 0 |  |  | 1 |  |  |  |
| Nitzschia exilis Sovereign | 8 | 0.026 |  |  |  |  |  |  | 8.928 |
| Nitzschia fonticola (Grunow) Grunow | 2 | 0.005 |  |  |  |  |  |  |  |
| Nitzschia frustulum (Kützing) Grunow | 1 | 0.005 | 0 |  |  | 1 |  |  |  |
| Nitzschia fruticosa Hustedt | 5 | 0.037 |  | 0 | 0 |  | 1 | 1 | 10.445 |
| Nitzschia gracilis Hantzsch | 52 | 0.823 |  | 0 | 0 |  | 1 | 1 | 10.561 |
| Nitzschia homburgiensis Lange-Bertalot | 1 | 0.003 |  |  |  |  |  |  |  |
| Nitzschia inconspicua Grunow | 2 | 0.005 |  |  |  |  |  |  | 9.383 |
| Nitzschia intermedia Hantzsch ex Cleve and Grunow | 9 | 0.038 | 0 |  |  | 1 |  |  | 8.684 |
| Nitzschia lacuum Lange-Bertalot | 1 | 0.003 |  |  |  |  |  |  | 8.460 |
| Nitzschia linearis (Agardh) W. Smith | 7 | 0.026 |  | 0 |  |  | 1 |  | 10.932 |
| Nitzschia nana Grunow ex Van Heurck | 40 | 0.786 | 0 | 0 | 0 | 1 | 1 | 1 | 9.629 |
| Nitzschia palea var. tenuirostris Grunow | 25 | 0.404 |  | 0 | 0 |  | 1 | 1 | 10.232 |
| Nitzschia paleacea Grunow | 7 | 0.037 |  |  |  |  |  |  | 9.402 |
| Nitzschia perminuta (Grunow) Peragallo | 11 | 0.121 | 0 |  |  | 1 |  |  | 9.316 |
| Nitzschia radicula Hustedt | 35 | 0.523 |  | 0 | 0 |  | 1 | 1 | 9.326 |
| Nitzschia semirobusta Lange-Bertalot | 8 | 0.056 |  |  |  |  |  |  | 8.739 |
| Nitzschia sociabilis Hustedt | 13 | 0.080 |  |  |  |  |  |  | 9.278 |
| Nitzschia subacicularis Hustedt | 31 | 0.340 |  | 0 | 0 |  | 1 | 1 | 9.105 |
| Nitzschia subtilis (Kützing) Grunow | 4 | 0.016 |  |  |  |  |  |  | 10.284 |
| Nitzschia wuellerstorffii Lange-Bertalot | 6 | 0.024 |  | 0 | 0 |  | 1 | 1 | 10.388 |
| Pinnularia | 34 | 0.648 |  | 0 | 0 |  | 1 | 1 | 10.213 |
| Pinnularia acrosphaeria (Brébisson) Smith | 19 | 0.106 |  | 0 | 0 |  | 1 | 1 | 10.394 |
| Pinnularia brebissonii (Kützing) Rabenhorst | 1 | 0.003 |  |  |  |  |  |  |  |
| Pinnularia gibba var. linearis Hustedt | 2 | 0.022 |  |  |  |  |  |  | 10.218 |
| Pinnularia microstauron (Ehrenberg) Cleve | 37 | 0.122 |  | 0 | 0 |  | 1 | 1 | 10.220 |
| Pinnularia obscura Krasske | 1 | 0.003 |  |  |  |  |  |  | 9.022 |
| Pinnularia subcapitata Gregory | 8 | 0.051 |  | 0 | 0 |  | 1 | 1 | 9.685 |
| Pinnularia subgibba Krammer | 1 | 0.002 |  |  |  |  |  |  |  |
| Pinnularia viridiformis Krammer | 7 | 0.015 |  |  |  |  |  |  | 6.846 |
| Pinnularia viridis (Nitzsch) Ehrenberg | 11 | 0.041 |  | 0 | 0 |  | 1 | 1 | 10.159 |
| Placoneis clementis (Grunow) Cox | 1 | 0.002 |  |  |  |  |  |  | 9.647 |
| Placoneis ignorata (Schimanski) Lange-Bertalot | 2 | 0.009 |  |  |  |  |  |  | 10.162 |
| Placoneis symmetrica (Hustedt) Lange-Bertalot | 1 | 0.006 |  |  |  |  |  |  | 10.353 |
| Planothidium lanceolatum (Brébisson ex Kützing) Lange-Bertalot | 1 | 0.002 |  |  |  |  |  |  |  |
| Psammothidium marginulatum (Grunow) Bukhtiyarova and Round | 1 | 0.002 |  |  |  |  |  |  | 11.764 |
| Rhopalodia | 1 | 0.003 |  |  |  |  |  |  |  |
| Rhopalodia gibba (Ehrenberg) Müller | 41 | 0.429 |  | 0 | 0 |  | 1 | 1 | 9.406 |
| Sellaphora | 7 | 0.061 |  | 0 |  |  | 1 |  | 11.818 |
| Sellaphora atomoides (Grunow) Wetzel and Van de Vijver | 6 | 0.024 |  | 0 |  |  | 1 |  | 10.588 |
| Sellaphora nigri (De Notaris) Wetzel and Ector | 3 | 0.017 |  |  |  |  |  |  | 10.649 |
| Sellaphora pupula (Kützing) Meresckowsky | 16 | 0.061 | 0 | 0 | 0 | 1 | 1 | 1 | 9.388 |
| Sellaphora rectangularis (Gregory) Lange-Bertalot and Metzeltin | 11 | 0.051 |  |  |  |  |  |  | 8.411 |
| Sellaphora saugerresii (Desmazières) Wetzel and Mann | 41 | 0.550 |  | 0 | 0 |  | 1 | 1 | 9.414 |
| Sellaphora wallacei (Reimer) Potapova and Ponader | 1 | 0.003 |  |  |  |  |  |  | 6.762 |
| Stauroneis | 4 | 0.007 |  |  |  |  |  |  | 9.500 |
| Stauroneis anceps Ehrenberg | 2 | 0.010 |  |  |  |  |  |  | 9.923 |
| Stauroneis gracilis Ehrenberg | 2 | 0.005 |  |  |  |  |  |  |  |
| Stauroneis obtusa Lagerstedt | 2 | 0.012 |  |  |  |  |  |  | 7.200 |
| Stauroneis phoenicenteron (Nitzsch) Ehrenberg | 3 | 0.008 |  |  |  |  |  |  | 8.908 |
| Stenopterobia curvula (Smith) Krammer | 4 | 0.030 |  |  |  |  |  |  | 9.573 |
| Stephanodiscus minutulus (Kützing) Cleve and Möller | 1 | 0.001 |  |  |  |  |  |  |  |
| Thalassiosira weissflogii (Grunow) G.A. Fryxell and Hasle | 1 | 0.004 |  |  |  |  |  |  | 11.044 |
| Ulnaria delicatissima var. angustissima (Grunow) Aboal | 34 | 0.389 |  | 0 | 0 |  | 1 | 1 | 10.002 |
| Ulnaria ulna (Nitzsch) Compére | 21 | 0.338 |  | 0 | 0 |  | 1 | 1 | 10.209 |
| Unknown (Undetermined pennate) | 3 | 0.020 |  |  |  |  |  |  | 6.391 |


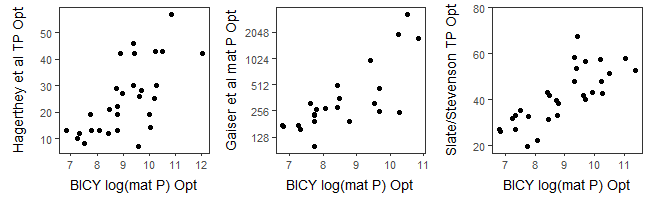


Fig. S5. Comparing mat and TP optimum traits across studies.

Table S4. Linear regression relationship of metrics with log transformed mat P. The models include the intercept, estimate of the slope, a standard error of the slope estimate, the p value for the statistical significance of the slope, a code for quick reference of p values (p*), and the adjusted R^2^ for relationships.

| Diversity or Trait-Based Metric | | | Intercept | Slope | | | | adj R2 |
| --- | --- | --- | --- | --- | --- | --- | --- | --- |
| Trait | Source | Metric.Type |  | Estimate | SE (est) | *p* | *p** |  |
| High P | Lit | noTaxa | -8.098 | 1.349 | 0.215 | <0.0001 | **** | 0.470 |
| High P | Lit | PropTaxa | -0.233 | 0.044 | 0.008 | <0.0001 | **** | 0.398 |
| High P | Lit | PropValves | -0.769 | 0.106 | 0.017 | <0.0001 | **** | 0.464 |
| High P | Lit | RlogA | -0.429 | 0.066 | 0.009 | <0.0001 | **** | 0.569 |
| High P | Regr | noTaxa | -19.241 | 3.427 | 0.422 | <0.0001 | **** | 0.601 |
| High P | Regr | PropTaxa | -0.347 | 0.095 | 0.012 | <0.0001 | **** | 0.600 |
| High P | Regr | PropValves | -1.088 | 0.171 | 0.025 | <0.0001 | **** | 0.523 |
| High P | Regr | RlogA | -0.657 | 0.128 | 0.015 | <0.0001 | **** | 0.638 |
| High P | TITAN | noTaxa | -18.032 | 3.519 | 0.464 | <0.0001 | **** | 0.568 |
| High P | TITAN | PropTaxa | -0.206 | 0.090 | 0.010 | <0.0001 | **** | 0.635 |
| High P | TITAN | PropValves | -1.073 | 0.178 | 0.023 | <0.0001 | **** | 0.574 |
| High P | TITAN | RlogA | -0.596 | 0.131 | 0.013 | <0.0001 | **** | 0.699 |
| Low P | Lit | noTaxa | 8.528 | -0.525 | 0.141 | 0.0006 | *** | 0.232 |
| Low P | Lit | PropTaxa | 0.745 | -0.061 | 0.009 | <0.0001 | **** | 0.531 |
| Low P | Lit | PropValves | 1.331 | -0.105 | 0.020 | <0.0001 | **** | 0.375 |
| Low P | Lit | RlogA | 0.971 | -0.079 | 0.010 | <0.0001 | **** | 0.607 |
| Low P | Regr | noTaxa | 12.919 | -0.909 | 0.153 | <0.0001 | **** | 0.444 |
| Low P | Regr | PropTaxa | 1.104 | -0.095 | 0.010 | <0.0001 | **** | 0.666 |
| Low P | Regr | PropValves | 2.078 | -0.182 | 0.024 | <0.0001 | **** | 0.572 |
| Low P | Regr | RlogA | 1.577 | -0.139 | 0.014 | <0.0001 | **** | 0.708 |
| Low P | TITAN | noTaxa | 14.111 | -1.017 | 0.160 | <0.0001 | **** | 0.477 |
| Low P | TITAN | PropTaxa | 1.187 | -0.103 | 0.010 | <0.0001 | **** | 0.701 |
| Low P | TITAN | PropValves | 2.082 | -0.182 | 0.024 | <0.0001 | **** | 0.573 |
| Low P | TITAN | RlogA | 1.610 | -0.142 | 0.013 | <0.0001 | **** | 0.720 |
| evenness | | diversity | 0.661 | -0.001 | 0.009 | 0.8828 |  | -0.023 |
| richness | | diversity | -7.171 | 3.230 | 0.623 | <0.0001 | **** | 0.376 |
| ShannonH | | diversity | 1.045 | 0.103 | 0.040 | 0.0142 | * | 0.114 |
| WAM_matP | | Inferred mat P | 4.744 | 0.426 | 0.051 | <0.0001 | **** | 0.619 |
| WAM_RlogA.matP | | Inferred mat P | 5.390 | 0.375 | 0.036 | <0.0001 | **** | 0.710 |

Table S5. Linear regression relationship of metrics and mat P with three independent variables (IndepVar): longitude, latitude, and lat*long interaction term. The regression results include an estimate of the intercept and slopes associated with each independent variable, as well as a standard error (SE (est)) of the slope estimate, the p value for the statistical significance of the slope, a code for quick reference of p values (p*), and the adjusted R^2^ for relationships.

| Metric or Mat P | | | IndepVar | Estimate | SE (est) | *p* | *p** | adj R2 |
| --- | --- | --- | --- | --- | --- | --- | --- | --- |
| Trait | Source | Metric.Type |  |  |  |  |  |  |
| High P | Lit | noTaxa | intercept | -5.169E+04 | 1.089E+04 | <0.0001 | **** |  |
| High P | Lit | noTaxa | longitude | 1.084E-01 | 2.292E-02 | <0.0001 | **** |  |
| High P | Lit | noTaxa | latitude | 1.791E-02 | 3.761E-03 | <0.0001 | **** |  |
| High P | Lit | noTaxa | lat*long | -3.760E-08 | 7.920E-09 | <0.0001 | **** | 0.5494 |
| High P | Lit | PropTaxa | intercept | -1.612E+03 | 4.120E+02 | 0.0003 | *** |  |
| High P | Lit | PropTaxa | longitude | 3.380E-03 | 8.673E-04 | 0.0004 | *** |  |
| High P | Lit | PropTaxa | latitude | 5.585E-04 | 1.423E-04 | 0.0003 | *** |  |
| High P | Lit | PropTaxa | lat*long | -1.170E-09 | 3.000E-10 | 0.0003 | *** | 0.4925 |
| High P | Lit | PropValves | intercept | -3.407E+03 | 9.309E+02 | 0.0007 | *** |  |
| High P | Lit | PropValves | longitude | 7.106E-03 | 1.960E-03 | 0.0008 | *** |  |
| High P | Lit | PropValves | latitude | 1.180E-03 | 3.217E-04 | 0.0007 | *** |  |
| High P | Lit | PropValves | lat*long | -2.460E-09 | 6.770E-10 | 0.0008 | *** | 0.4776 |
| High P | Lit | RlogA | intercept | -2.297E+03 | 4.277E+02 | <0.0001 | **** |  |
| High P | Lit | RlogA | longitude | 4.802E-03 | 9.004E-04 | <0.0001 | **** |  |
| High P | Lit | RlogA | latitude | 7.956E-04 | 1.478E-04 | <0.0001 | **** |  |
| High P | Lit | RlogA | lat*long | -1.660E-09 | 3.110E-10 | <0.0001 | **** | 0.6533 |
| High P | Regr | noTaxa | intercept | -1.094E+05 | 2.423E+04 | 0.0001 | **** |  |
| High P | Regr | noTaxa | longitude | 2.290E-01 | 5.102E-02 | 0.0001 | **** |  |
| High P | Regr | noTaxa | latitude | 3.791E-02 | 8.373E-03 | 0.0001 | **** |  |
| High P | Regr | noTaxa | lat*long | -7.940E-08 | 1.760E-08 | 0.0001 | **** | 0.5624 |
| High P | Regr | PropTaxa | intercept | -2.514E+03 | 7.400E+02 | 0.0016 | ** |  |
| High P | Regr | PropTaxa | longitude | 5.249E-03 | 1.558E-03 | 0.0017 | ** |  |
| High P | Regr | PropTaxa | latitude | 8.712E-04 | 2.557E-04 | 0.0015 | ** |  |
| High P | Regr | PropTaxa | lat*long | -1.820E-09 | 5.380E-10 | 0.0016 | ** | 0.4690 |
| High P | Regr | PropValves | intercept | -5.132E+03 | 1.296E+03 | 0.0003 | *** |  |
| High P | Regr | PropValves | longitude | 1.072E-02 | 2.728E-03 | 0.0003 | *** |  |
| High P | Regr | PropValves | latitude | 1.779E-03 | 4.478E-04 | 0.0003 | *** |  |
| High P | Regr | PropValves | lat*long | -3.710E-09 | 9.430E-10 | 0.0003 | *** | 0.5613 |
| High P | Regr | RlogA | intercept | -3.328E+03 | 8.677E+02 | 0.0004 | *** |  |
| High P | Regr | RlogA | longitude | 6.942E-03 | 1.827E-03 | 0.0005 | *** |  |
| High P | Regr | RlogA | latitude | 1.154E-03 | 2.998E-04 | 0.0004 | *** |  |
| High P | Regr | RlogA | lat*long | -2.410E-09 | 6.310E-10 | 0.0005 | *** | 0.5771 |
| High P | TITAN | noTaxa | intercept | -1.078E+05 | 2.668E+04 | 0.0002 | *** |  |
| High P | TITAN | noTaxa | longitude | 2.258E-01 | 5.616E-02 | 0.0003 | *** |  |
| High P | TITAN | noTaxa | latitude | 3.737E-02 | 9.217E-03 | 0.0002 | *** |  |
| High P | TITAN | noTaxa | lat*long | -7.820E-08 | 1.940E-08 | 0.0002 | *** | 0.5244 |
| High P | TITAN | PropTaxa | intercept | -1.918E+03 | 6.953E+02 | 0.0087 | ** |  |
| High P | TITAN | PropTaxa | longitude | 3.998E-03 | 1.464E-03 | 0.0093 | ** |  |
| High P | TITAN | PropTaxa | latitude | 6.654E-04 | 2.402E-04 | 0.0085 | ** |  |
| High P | TITAN | PropTaxa | lat*long | -1.390E-09 | 5.060E-10 | 0.0091 | ** | 0.4478 |
| High P | TITAN | PropValves | intercept | -4.107E+03 | 1.409E+03 | 0.0058 | ** |  |
| High P | TITAN | PropValves | longitude | 8.562E-03 | 2.967E-03 | 0.0063 | ** |  |
| High P | TITAN | PropValves | latitude | 1.424E-03 | 4.870E-04 | 0.0057 | ** |  |
| High P | TITAN | PropValves | lat*long | -2.970E-09 | 1.030E-09 | 0.0061 | ** | 0.4778 |
| High P | TITAN | RlogA | intercept | -2.674E+03 | 9.391E+02 | 0.0069 | ** |  |
| High P | TITAN | RlogA | longitude | 5.567E-03 | 1.977E-03 | 0.0075 | ** |  |
| High P | TITAN | RlogA | latitude | 9.275E-04 | 3.245E-04 | 0.0067 | ** |  |
| High P | TITAN | RlogA | lat*long | -1.930E-09 | 6.830E-10 | 0.0073 | ** | 0.4830 |
| Low P | Lit | noTaxa | intercept | 1.642E+04 | 7.701E+03 | 0.0391 | * |  |
| Low P | Lit | noTaxa | longitude | -3.411E-02 | 1.621E-02 | 0.0417 | * |  |
| Low P | Lit | noTaxa | latitude | -5.678E-03 | 2.661E-03 | 0.0390 | * |  |
| Low P | Lit | noTaxa | lat*long | 1.180E-08 | 5.600E-09 | 0.0415 | * | 0.2322 |
| Low P | Lit | PropTaxa | intercept | 1.783E+03 | 5.186E+02 | 0.0014 | ** |  |
| Low P | Lit | PropTaxa | longitude | -3.725E-03 | 1.092E-03 | 0.0015 | ** |  |
| Low P | Lit | PropTaxa | latitude | -6.176E-04 | 1.792E-04 | 0.0013 | ** |  |
| Low P | Lit | PropTaxa | lat*long | 1.290E-09 | 3.770E-10 | 0.0015 | ** | 0.4361 |
| Low P | Lit | PropValves | intercept | 3.618E+03 | 1.085E+03 | 0.0018 | ** |  |
| Low P | Lit | PropValves | longitude | -7.575E-03 | 2.284E-03 | 0.0019 | ** |  |
| Low P | Lit | PropValves | latitude | -1.253E-03 | 3.748E-04 | 0.0018 | ** |  |
| Low P | Lit | PropValves | lat*long | 2.620E-09 | 7.890E-10 | 0.0019 | ** | 0.4007 |
| Low P | Lit | RlogA | intercept | 2.399E+03 | 5.731E+02 | 0.0002 | *** |  |
| Low P | Lit | RlogA | longitude | -5.011E-03 | 1.206E-03 | 0.0002 | *** |  |
| Low P | Lit | RlogA | latitude | -8.308E-04 | 1.980E-04 | 0.0001 | *** |  |
| Low P | Lit | RlogA | lat*long | 1.740E-09 | 4.170E-10 | 0.0002 | *** | 0.5330 |
| Low P | Regr | noTaxa | intercept | 2.114E+04 | 9.400E+03 | 0.0301 | * |  |
| Low P | Regr | noTaxa | longitude | -4.395E-02 | 1.979E-02 | 0.0321 | * |  |
| Low P | Regr | noTaxa | latitude | -7.320E-03 | 3.248E-03 | 0.0298 | * |  |
| Low P | Regr | noTaxa | lat*long | 1.520E-08 | 6.840E-09 | 0.0317 | * | 0.2991 |
| Low P | Regr | PropTaxa | intercept | 2.306E+03 | 7.250E+02 | 0.0028 | ** |  |
| Low P | Regr | PropTaxa | longitude | -4.818E-03 | 1.526E-03 | 0.0030 | ** |  |
| Low P | Regr | PropTaxa | latitude | -7.991E-04 | 2.505E-04 | 0.0028 | ** |  |
| Low P | Regr | PropTaxa | lat*long | 1.670E-09 | 5.270E-10 | 0.0029 | ** | 0.4399 |
| Low P | Regr | PropValves | intercept | 4.072E+03 | 1.456E+03 | 0.0079 | ** |  |
| Low P | Regr | PropValves | longitude | -8.490E-03 | 3.066E-03 | 0.0085 | ** |  |
| Low P | Regr | PropValves | latitude | -1.412E-03 | 5.031E-04 | 0.0077 | ** |  |
| Low P | Regr | PropValves | lat*long | 2.940E-09 | 1.060E-09 | 0.0082 | ** | 0.4642 |
| Low P | Regr | RlogA | intercept | 2.936E+03 | 9.985E+02 | 0.0054 | ** |  |
| Low P | Regr | RlogA | longitude | -6.119E-03 | 2.102E-03 | 0.0059 | ** |  |
| Low P | Regr | RlogA | latitude | -1.018E-03 | 3.450E-04 | 0.0053 | ** |  |
| Low P | Regr | RlogA | lat*long | 2.120E-09 | 7.260E-10 | 0.0057 | ** | 0.4715 |
| Low P | TITAN | noTaxa | intercept | 2.340E+04 | 9.873E+03 | 0.0227 | * |  |
| Low P | TITAN | noTaxa | longitude | -4.862E-02 | 2.079E-02 | 0.0244 | * |  |
| Low P | TITAN | noTaxa | latitude | -8.102E-03 | 3.411E-03 | 0.0224 | * |  |
| Low P | TITAN | noTaxa | lat*long | 1.680E-08 | 7.180E-09 | 0.0241 | * | 0.3386 |
| Low P | TITAN | PropTaxa | intercept | 2.481E+03 | 7.373E+02 | 0.0017 | ** |  |
| Low P | TITAN | PropTaxa | longitude | -5.183E-03 | 1.552E-03 | 0.0018 | ** |  |
| Low P | TITAN | PropTaxa | latitude | -8.599E-04 | 2.548E-04 | 0.0016 | ** |  |
| Low P | TITAN | PropTaxa | lat*long | 1.800E-09 | 5.360E-10 | 0.0018 | ** | 0.4825 |
| Low P | TITAN | PropValves | intercept | 4.086E+03 | 1.454E+03 | 0.0076 | ** |  |
| Low P | TITAN | PropValves | longitude | -8.520E-03 | 3.062E-03 | 0.0082 | ** |  |
| Low P | TITAN | PropValves | latitude | -1.417E-03 | 5.025E-04 | 0.0074 | ** |  |
| Low P | TITAN | PropValves | lat*long | 2.960E-09 | 1.060E-09 | 0.0080 | ** | 0.4661 |
| Low P | TITAN | RlogA | intercept | 3.030E+03 | 9.975E+02 | 0.0042 | ** |  |
| Low P | TITAN | RlogA | longitude | -6.317E-03 | 2.100E-03 | 0.0045 | ** |  |
| Low P | TITAN | RlogA | latitude | -1.051E-03 | 3.446E-04 | 0.0041 | ** |  |
| Low P | TITAN | RlogA | lat*long | 2.190E-09 | 7.260E-10 | 0.0044 | ** | 0.4869 |
| evenness | | diversity | intercept | -3.301E+02 | 5.097E+02 | 0.5210 |  |  |
| evenness | | diversity | longitude | 7.072E-04 | 1.073E-03 | 0.5137 |  |  |
| evenness | | diversity | latitude | 1.144E-04 | 1.761E-04 | 0.5198 |  |  |
| evenness | | diversity | lat*long | -2.450E-10 | 3.710E-10 | 0.5133 |  | -0.0279 |
| richness |  | diversity | intercept | -1.109E+05 | 3.428E+04 | 0.0024 | ** |  |
| richness |  | diversity | longitude | 2.328E-01 | 7.216E-02 | 0.0025 | ** |  |
| richness |  | diversity | latitude | 3.843E-02 | 1.184E-02 | 0.0024 | ** |  |
| richness |  | diversity | lat*long | -8.070E-08 | 2.490E-08 | 0.0024 | ** | 0.3700 |
| ShannonH | | diversity | intercept | -4.311E+03 | 2.207E+03 | 0.0578 |  |  |
| ShannonH | | diversity | longitude | 9.088E-03 | 4.646E-03 | 0.0575 |  |  |
| ShannonH | | diversity | latitude | 1.494E-03 | 7.626E-04 | 0.0571 |  |  |
| ShannonH | | diversity | lat*long | -3.150E-09 | 1.610E-09 | 0.0568 |  | 0.1191 |
| WAM_matP | | Inferred mat P | intercept | -1.116E+04 | 2.950E+03 | 0.0005 | *** |  |
| WAM_matP | | Inferred mat P | longitude | 2.328E-02 | 6.210E-03 | 0.0006 | *** |  |
| WAM_matP | | Inferred mat P | latitude | 3.870E-03 | 1.019E-03 | 0.0005 | *** |  |
| WAM_matP | | Inferred mat P | lat*long | -8.070E-09 | 2.150E-09 | 0.0005 | *** | 0.5696 |
| WAM_RlogA.matP | | Inferred mat P | intercept | -9.533E+03 | 2.361E+03 | 0.0002 | *** |  |
| WAM_RlogA.matP | | Inferred mat P | longitude | 1.989E-02 | 4.970E-03 | 0.0003 | *** |  |
| WAM_RlogA.matP | | Inferred mat P | latitude | 3.306E-03 | 8.157E-04 | 0.0002 | *** |  |
| WAM_RlogA.matP | | Inferred mat P | lat*long | -6.890E-09 | 1.720E-09 | 0.0003 | *** | 0.5939 |
| mat P |  |  | intercept | -8.247E+06 | 2.233E+06 | 0.0007 | *** |  |
| mat P |  |  | longitude | 1.722E+00 | 4.700E+00 | 0.0007 | *** |  |
| mat P |  |  | latitude | 2.855E+00 | 7.714E-01 | 0.0006 | *** |  |
| mat P |  |  | lat*long | -5.960E-06 | 1.624E-06 | 0.0007 | *** | 0.4375 |

Table S6. ANOVA results for effects of BCNP region on metrics. The ANOVA results include an F value as well as the p value for attained statistical significance and a code for quick reference of p values (p*).

| Diversity or Trait-Based Metric | | | F value | p | p* |
| --- | --- | --- | --- | --- | --- |
| Trait | Source | Metric.Type |  |  |  |
| High P | Lit | noTaxa | 20.5 | <0.0001 | **** |
| High P | Lit | PropTaxa | 10.6 | <0.0001 | **** |
| High P | Lit | PropValves | 13.1 | <0.0001 | **** |
| High P | Lit | RlogA | 16.1 | <0.0001 | **** |
| High P | Regr | noTaxa | 22.6 | <0.0001 | **** |
| High P | Regr | PropTaxa | 13.6 | <0.0001 | **** |
| High P | Regr | PropValves | 22.2 | <0.0001 | **** |
| High P | Regr | RlogA | 18.5 | <0.0001 | **** |
| High P | TITAN | noTaxa | 26.8 | <0.0001 | **** |
| High P | TITAN | PropTaxa | 17.5 | <0.0001 | **** |
| High P | TITAN | PropValves | 13.3 | <0.0001 | **** |
| High P | TITAN | RlogA | 19.9 | <0.0001 | **** |
| Low P | Lit | noTaxa | 8.7 | <0.0001 | **** |
| Low P | Lit | PropTaxa | 12.1 | <0.0001 | **** |
| Low P | Lit | PropValves | 10.8 | <0.0001 | **** |
| Low P | Lit | RlogA | 22.0 | <0.0001 | **** |
| Low P | Regr | noTaxa | 22.6 | <0.0001 | **** |
| Low P | Regr | PropTaxa | 17.1 | <0.0001 | **** |
| Low P | Regr | PropValves | 16.5 | <0.0001 | **** |
| Low P | Regr | RlogA | 21.3 | <0.0001 | **** |
| Low P | TITAN | noTaxa | 20.3 | <0.0001 | **** |
| Low P | TITAN | PropTaxa | 18.6 | <0.0001 | **** |
| Low P | TITAN | PropValves | 16.4 | <0.0001 | **** |
| Low P | TITAN | RlogA | 21.1 | <0.0001 | **** |
| richness | | diversity | 18.2 | <0.0001 | **** |
| evenness | | diversity | 2.4 | 0.0472 | * |
| ShannonH | | diversity | 8.3 | <0.0001 | **** |
| WAM_matP | | Inferred mat P | 30.5 | <0.0001 | **** |
| WAM_RlogA.matP | | Inferred mat P | 29.1 | <0.0001 | **** |


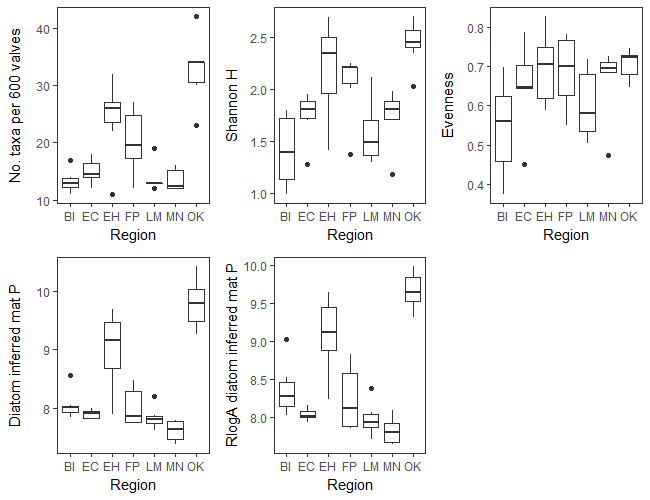


Fig. S6a. Boxplots of diversity and weighted average metrics for regions in BCNP sampling area. BI = Bear Island, EC = East Crossing, EH =East Hinson Marsh, FP = Fire Prairie, LM = Little Marsh, MN = Monument, and OK = Okaloacoochee Slough.


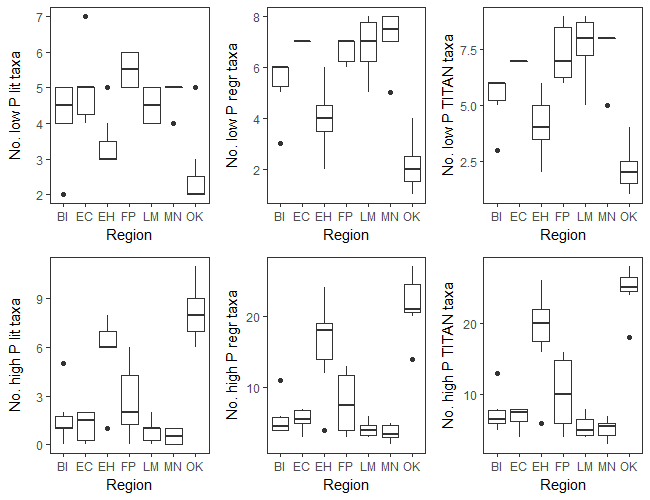


Fig. S6b. Boxplots of metrics based on the number of taxa for regions in BCNP sampling area. Region codes correspond to region names in Fig. S6a.


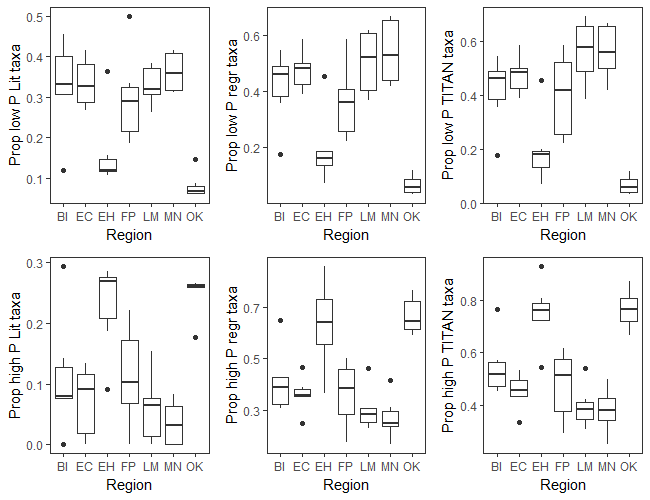


Fig. S6c. Boxplots of metrics based on the proportion of taxa for regions in BCNP sampling area. Region codes correspond to region names in Fig. S6a.


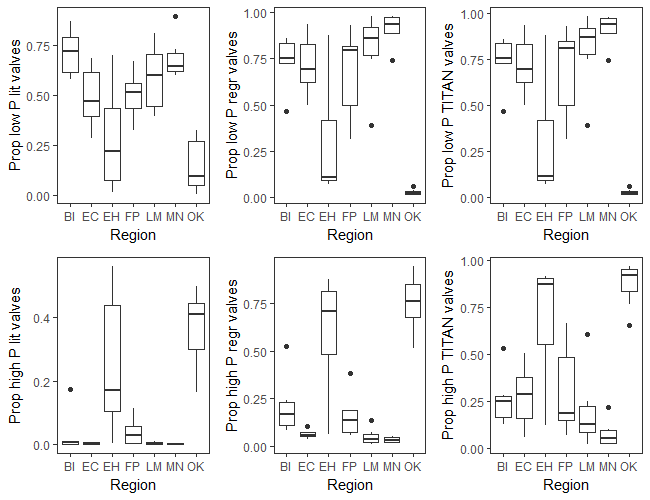


Fig. S6d. Boxplots of metrics based on proportion of valves for regions in BCNP sampling area. Region codes correspond to region names in Fig. S6a.


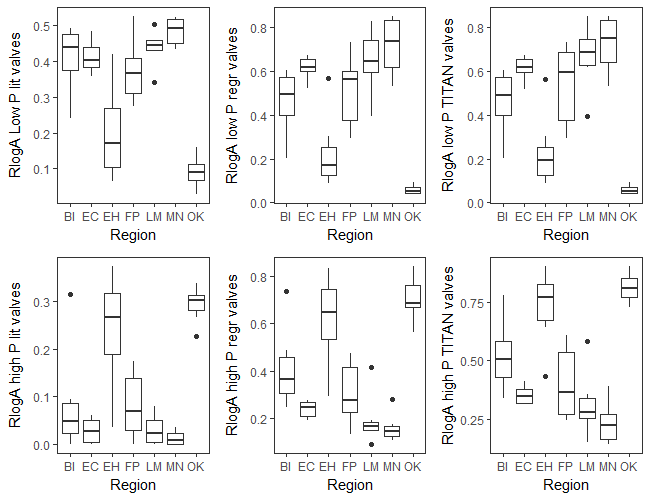


Fig. S6e. Boxplots of metrics based on RlogA valves for regions in BCNP sampling area. Region codes correspond to region names in Fig. S6a.


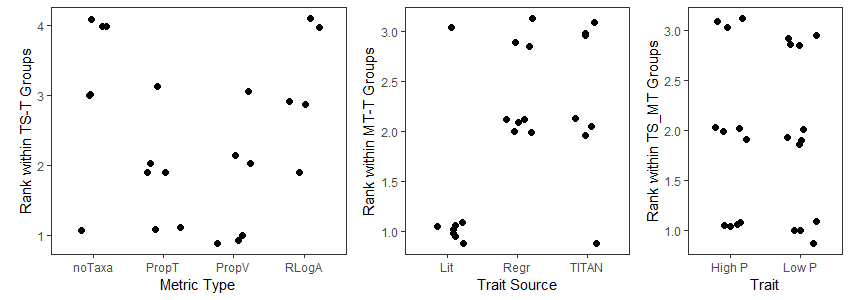


Fig. S7. Plots of the ranks of F statistics for each ANOVA evaluating effects of region on metrics to show effects of metric type, trait source, and trait on assessments of differences in metrics among regions using rank of F values for metric - region ANOVA for metric type (ranks 1-4) within trait-source-trait groups, for trait source (ranks 1-3) within metric type-trait groups, and trait (ranks 1-2) within trait source-metric type groups.

Table S7. Linear regression relationship of residuals from metric-mat P relationships with the independent variables (IndVar) depth and season. The regression results include an estimate of the intercept and slopes associated with each independent variable, as well as a standard error (SE (est)) of the slope estimate, the p value for the statistical significance of the slope, a code for quick reference of p values (p*), and the adjusted R^2^ for relationships.

| IndVar | Diversity or Trait-Based Metric | | | Intercept | Slope | | | | adj R2 |
| --- | --- | --- | --- | --- | --- | --- | --- | --- | --- |
|  | Trait | Source | Metric.Type |  | Estimate | SE (est) | *p* | *p** |  |
| Depth | High P | Lit | noTaxa | -0.133 | 0.016 | 0.023 | 0.4873 |  | -0.015 |
| Depth | High P | Lit | PropTaxa | -0.025 | 0.001 | 0.001 | 0.1277 |  | 0.041 |
| Depth | High P | Lit | PropValves | -0.012 | 0.001 | 0.002 | 0.4778 |  | -0.014 |
| Depth | High P | Lit | RlogA | -0.017 | 0.001 | 0.001 | 0.1943 |  | 0.022 |
| Depth | High P | Regr | noTaxa | 1.321 | -0.035 | 0.048 | 0.4715 |  | -0.014 |
| Depth | High P | Regr | PropTaxa | 0.012 | 0.000 | 0.001 | 0.8686 |  | -0.029 |
| Depth | High P | Regr | PropValves | 0.005 | 0.001 | 0.002 | 0.7806 |  | -0.028 |
| Depth | High P | Regr | RlogA | -0.002 | 0.001 | 0.002 | 0.7589 |  | -0.027 |
| Depth | High P | TITAN | noTaxa | 1.198 | -0.035 | 0.053 | 0.5228 |  | -0.017 |
| Depth | High P | TITAN | PropTaxa | -0.011 | 0.000 | 0.001 | 0.6836 |  | -0.025 |
| Depth | High P | TITAN | PropValves | -0.011 | 0.001 | 0.002 | 0.7339 |  | -0.027 |
| Depth | High P | TITAN | RlogA | -0.023 | 0.001 | 0.001 | 0.4962 |  | -0.016 |
| Depth | Low P | Lit | noTaxa | -0.059 | 0.000 | 0.017 | 0.9771 |  | -0.030 |
| Depth | Low P | Lit | PropTaxa | -0.025 | 0.001 | 0.001 | 0.3859 |  | -0.007 |
| Depth | Low P | Lit | PropValves | 0.013 | -0.001 | 0.002 | 0.6293 |  | -0.023 |
| Depth | Low P | Lit | RlogA | -0.013 | 0.000 | 0.001 | 0.8139 |  | -0.029 |
| Depth | Low P | Regr | noTaxa | 0.018 | 0.000 | 0.018 | 0.9885 |  | -0.030 |
| Depth | Low P | Regr | PropTaxa | -0.025 | 0.001 | 0.001 | 0.3221 |  | 0.000 |
| Depth | Low P | Regr | PropValves | 0.004 | 0.000 | 0.002 | 0.8737 |  | -0.030 |
| Depth | Low P | Regr | RlogA | -0.001 | 0.000 | 0.001 | 0.9108 |  | -0.030 |
| Depth | Low P | TITAN | noTaxa | 0.008 | 0.001 | 0.019 | 0.9618 |  | -0.030 |
| Depth | Low P | TITAN | PropTaxa | -0.026 | 0.001 | 0.001 | 0.2769 |  | 0.006 |
| Depth | Low P | TITAN | PropValves | 0.004 | 0.000 | 0.002 | 0.8784 |  | -0.030 |
| Depth | Low P | TITAN | RlogA | -0.001 | 0.000 | 0.001 | 0.8876 |  | -0.030 |
| Depth | evenness | | diversity | 2.257 | -0.077 | 0.073 | 0.3039 |  | 0.003 |
| Depth | richness | | diversity | 0.006 | 0.000 | 0.001 | 0.9033 |  | -0.030 |
| Depth | ShannonH | | diversity | 0.084 | -0.003 | 0.005 | 0.5748 |  | -0.020 |
| Depth | WAM_matP | | Inferred mat P | -0.062 | 0.005 | 0.005 | 0.3867 |  | -0.007 |
| Depth | WAM_RlogA.matP | | Inferred mat P | -0.018 | 0.002 | 0.004 | 0.6362 |  | -0.023 |
| Season | High P | Lit | noTaxa | 1.562 | -0.486 | 0.143 | 0.0015 | ** | 0.197 |
| Season | High P | Lit | PropTaxa | 0.027 | -0.008 | 0.006 | 0.1694 |  | 0.022 |
| Season | High P | Lit | PropValves | -0.012 | 0.004 | 0.013 | 0.7787 |  | -0.022 |
| Season | High P | Lit | RlogA | 0.024 | -0.008 | 0.006 | 0.2476 |  | 0.009 |
| Season | High P | Regr | noTaxa | 2.163 | -0.673 | 0.299 | 0.0298 | * | 0.086 |
| Season | High P | Regr | PropTaxa | -0.032 | 0.010 | 0.009 | 0.2618 |  | 0.007 |
| Season | High P | Regr | PropValves | 0.017 | -0.005 | 0.018 | 0.7763 |  | -0.022 |
| Season | High P | Regr | RlogA | -0.021 | 0.006 | 0.011 | 0.5576 |  | -0.015 |
| Season | High P | TITAN | noTaxa | 2.977 | -0.927 | 0.318 | 0.0056 | ** | 0.149 |
| Season | High P | TITAN | PropTaxa | -0.022 | 0.007 | 0.008 | 0.3837 |  | -0.005 |
| Season | High P | TITAN | PropValves | -0.032 | 0.010 | 0.017 | 0.5698 |  | -0.016 |
| Season | High P | TITAN | RlogA | -0.028 | 0.009 | 0.010 | 0.3731 |  | -0.004 |
| Season | Low P | Lit | noTaxa | 0.620 | -0.193 | 0.101 | 0.0634 |  | 0.058 |
| Season | Low P | Lit | PropTaxa | 0.005 | -0.001 | 0.006 | 0.8295 |  | -0.023 |
| Season | Low P | Lit | PropValves | -0.037 | 0.012 | 0.015 | 0.4470 |  | -0.010 |
| Season | Low P | Lit | RlogA | -0.001 | 0.000 | 0.007 | 0.9592 |  | -0.024 |
| Season | Low P | Regr | noTaxa | 0.700 | -0.218 | 0.110 | 0.0532 |  | 0.064 |
| Season | Low P | Regr | PropTaxa | 0.008 | -0.002 | 0.008 | 0.7525 |  | -0.021 |
| Season | Low P | Regr | PropValves | 0.032 | -0.010 | 0.018 | 0.5810 |  | -0.016 |
| Season | Low P | Regr | RlogA | 0.022 | -0.007 | 0.010 | 0.4981 |  | -0.013 |
| Season | Low P | TITAN | noTaxa | 0.675 | -0.210 | 0.116 | 0.0769 |  | 0.051 |
| Season | Low P | TITAN | PropTaxa | 0.002 | -0.001 | 0.008 | 0.9336 |  | -0.024 |
| Season | Low P | TITAN | PropValves | 0.032 | -0.010 | 0.018 | 0.5761 |  | -0.016 |
| Season | Low P | TITAN | RlogA | 0.021 | -0.007 | 0.010 | 0.5118 |  | -0.013 |
| Season | evenness | | diversity | 4.769 | -1.484 | 0.407 | 0.0007 | *** | 0.222 |
| Season | richness | | diversity | 0.044 | -0.014 | 0.007 | 0.0447 | * | 0.071 |
| Season | ShannonH | | diversity | 0.263 | -0.082 | 0.028 | 0.0049 | ** | 0.154 |
| Season | WAM_matP | | Inferred mat P | -0.104 | 0.032 | 0.038 | 0.3946 |  | -0.006 |
| Season | WAM_RlogA.matP | | Inferred mat P | -0.041 | 0.013 | 0.027 | 0.6422 |  | -0.018 |

Table S8. ANOVA results for effects of habitat type, substrate location, substrate type, and hydrologic year on residuals in metric-mat P relatinships. The ANOVA results include an F value as well as the p value for attained statistical significance and a code for quick reference of p values (p*).

| Diversity or Trait-Based Metric | | | Habitat Type | | | Substrate Location | | | Substrate Type | | | Hydrological Year | | |
| --- | --- | --- | --- | --- | --- | --- | --- | --- | --- | --- | --- | --- | --- | --- |
| Trait | Source | Metric.Type | F value | p | p* | F value | p | p* | F value | p | p* | F.value | p | p* |
| High P | Lit | noTaxa | 0.6 | 0.7292 |  | 1.4 | 0.2518 |  | 1.1 | 0.3417 |  | 1.4 | 0.2404 |  |
| High P | Lit | PropTaxa | 1.2 | 0.3505 |  | 0.3 | 0.7521 |  | 0.9 | 0.4112 |  | 0.0 | 0.9661 |  |
| High P | Lit | PropValves | 5.1 | 0.0011 | ** | 3.1 | 0.0561 |  | 5.9 | 0.0057 | ** | 0.3 | 0.5864 |  |
| High P | Lit | RlogA | 3.0 | 0.0238 | * | 2.3 | 0.1167 |  | 2.9 | 0.0637 |  | 0.0 | 0.8912 |  |
| High P | Regr | noTaxa | 1.4 | 0.2586 |  | 2.0 | 0.1473 |  | 2.1 | 0.1379 |  | 0.6 | 0.4363 |  |
| High P | Regr | PropTaxa | 1.0 | 0.4239 |  | 2.4 | 0.1071 |  | 2.1 | 0.1303 |  | 2.4 | 0.1313 |  |
| High P | Regr | PropValves | 2.0 | 0.098 |  | 1.7 | 0.2045 |  | 2.3 | 0.1105 |  | 1.4 | 0.2354 |  |
| High P | Regr | RlogA | 1.9 | 0.1263 |  | 2.4 | 0.1023 |  | 2.1 | 0.1402 |  | 2.4 | 0.1282 |  |
| High P | TITAN | noTaxa | 1.1 | 0.3705 |  | 1.8 | 0.1757 |  | 0.2 | 0.8016 |  | 1.5 | 0.2312 |  |
| High P | TITAN | PropTaxa | 1.0 | 0.4215 |  | 0.3 | 0.7091 |  | 0.0 | 0.9532 |  | 0.4 | 0.5386 |  |
| High P | TITAN | PropValves | 1.4 | 0.2391 |  | 0.8 | 0.4585 |  | 1.7 | 0.2006 |  | 1.3 | 0.2617 |  |
| High P | TITAN | RlogA | 1.2 | 0.3504 |  | 0.5 | 0.6182 |  | 0.2 | 0.8003 |  | 1.1 | 0.2956 |  |
| Low P | Lit | noTaxa | 3.0 | 0.0232 | * | 1.3 | 0.2854 |  | 1.9 | 0.1608 |  | 1.5 | 0.2264 |  |
| Low P | Lit | PropTaxa | 1.0 | 0.4477 |  | 0.0 | 0.973 |  | 0.1 | 0.8771 |  | 1.3 | 0.2524 |  |
| Low P | Lit | PropValves | 1.3 | 0.2823 |  | 0.5 | 0.6222 |  | 4.2 | 0.022 | * | 0.0 | 0.9216 |  |
| Low P | Lit | RlogA | 1.7 | 0.1551 |  | 0.2 | 0.8248 |  | 1.6 | 0.2081 |  | 0.5 | 0.5036 |  |
| Low P | Regr | noTaxa | 2.7 | 0.0341 | * | 1.7 | 0.1965 |  | 1.6 | 0.2184 |  | 4.5 | 0.0397 | * |
| Low P | Regr | PropTaxa | 0.4 | 0.8453 |  | 0.3 | 0.7073 |  | 0.2 | 0.821 |  | 4.6 | 0.0382 | * |
| Low P | Regr | PropValves | 1.4 | 0.2427 |  | 0.5 | 0.6092 |  | 1.7 | 0.192 |  | 1.6 | 0.2181 |  |
| Low P | Regr | RlogA | 0.9 | 0.5111 |  | 0.0 | 0.9762 |  | 0.4 | 0.6919 |  | 2.5 | 0.1225 |  |
| Low P | TITAN | noTaxa | 2.0 | 0.1086 |  | 1.7 | 0.201 |  | 1.2 | 0.3014 |  | 5.6 | 0.0230 | * |
| Low P | TITAN | PropTaxa | 0.3 | 0.9181 |  | 0.1 | 0.8636 |  | 0.2 | 0.8071 |  | 6.0 | 0.0182 | * |
| Low P | TITAN | PropValves | 1.4 | 0.2444 |  | 0.5 | 0.6045 |  | 1.7 | 0.1915 |  | 1.6 | 0.2128 |  |
| Low P | TITAN | RlogA | 0.8 | 0.5624 |  | 0.0 | 0.955 |  | 0.4 | 0.7007 |  | 3.0 | 0.0930 |  |
| richness | | diversity | 1.5 | 0.2106 |  | 2.6 | 0.0847 |  | 0.1 | 0.949 |  | 1.6 | 0.2146 |  |
| evenness | | diversity | 1.8 | 0.1374 |  | 0.7 | 0.5047 |  | 0.1 | 0.9356 |  | 0.6 | 0.4597 |  |
| ShannonH | | diversity | 2.0 | 0.1083 |  | 1.2 | 0.305 |  | 0.1 | 0.9028 |  | 1.0 | 0.3217 |  |
| WAM_matP | | Inferred mat P | 6.0 | 0.0004 | *** | 3.2 | 0.0492 | * | 6.0 | 0.0053 | ** | 0.3 | 0.6003 |  |
| WAM_RlogA.matP | | Inferred mat P | 3.2 | 0.0158 | * | 2.2 | 0.1191 |  | 2.6 | 0.0896 |  | 0.7 | 0.3965 |  |


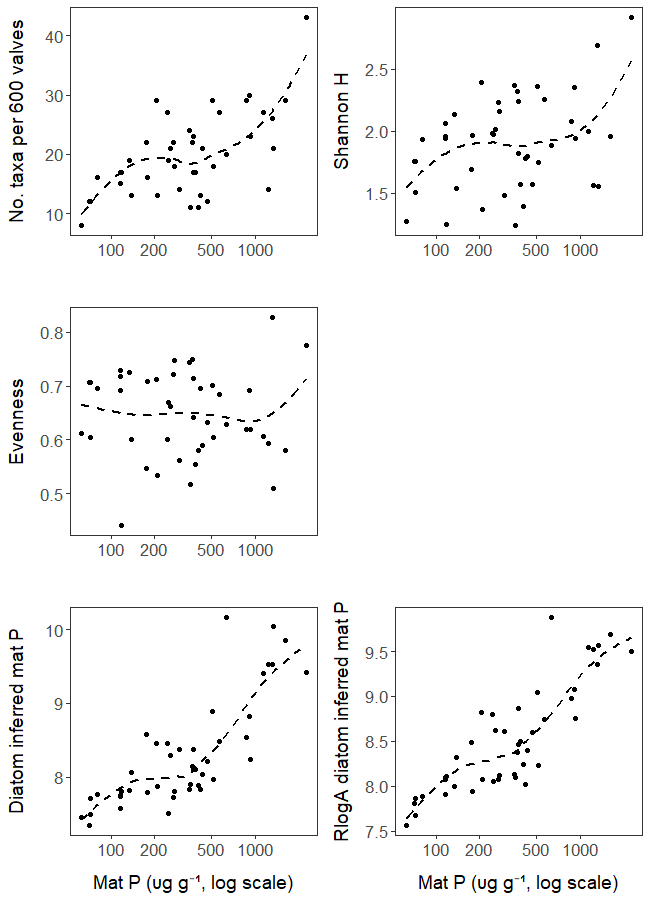


Fig. S8a. Relationships between diversity and weighted average metrics versus log-transformed mat P. The dashed black line represents a lowess fit to the data.


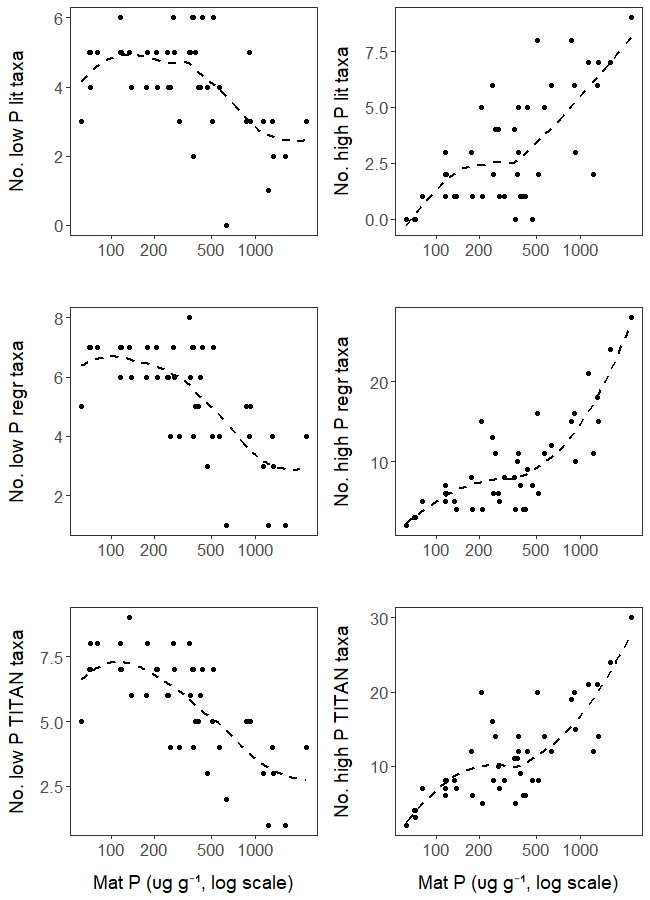


Fig. S8b. Relationships between metrics based on number of taxa versus log-transformed mat P. The dashed black line represents a lowess fit to the data.


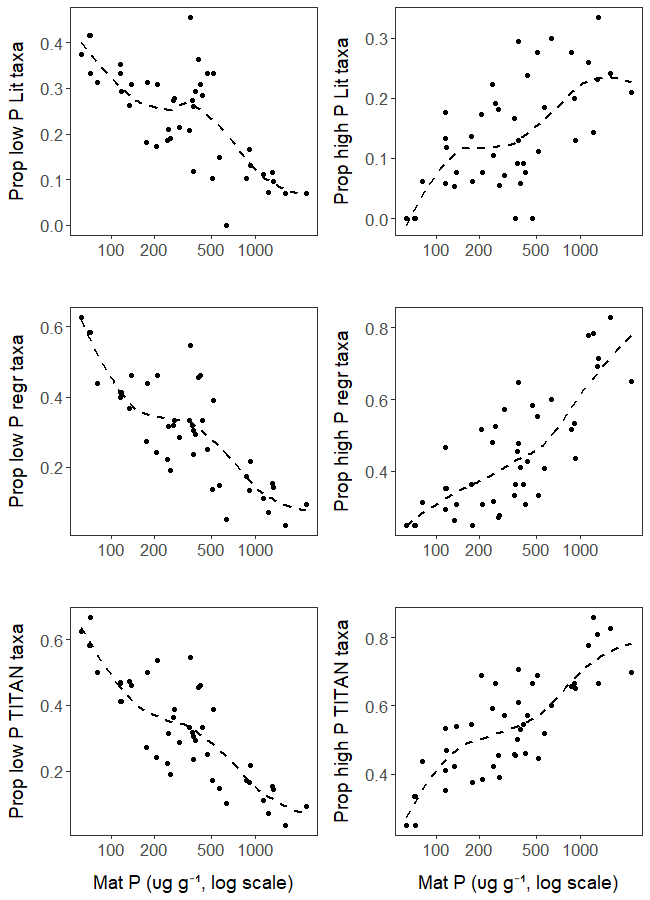


Fig. S8c. Relationships between metrics based on the proportion of taxa versus log-transformed mat P. The dashed black line represents a lowess fit to the data.


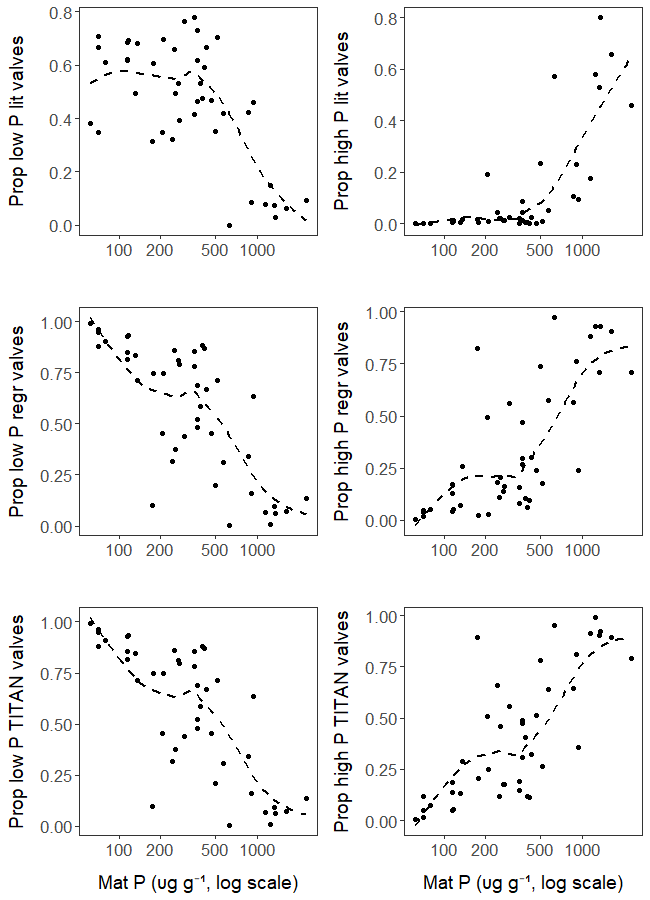


Fig. S8d. Relationships between metrics based on proportion of valves versus log-transformed mat P. The dashed black line represents a lowess fit to the data.


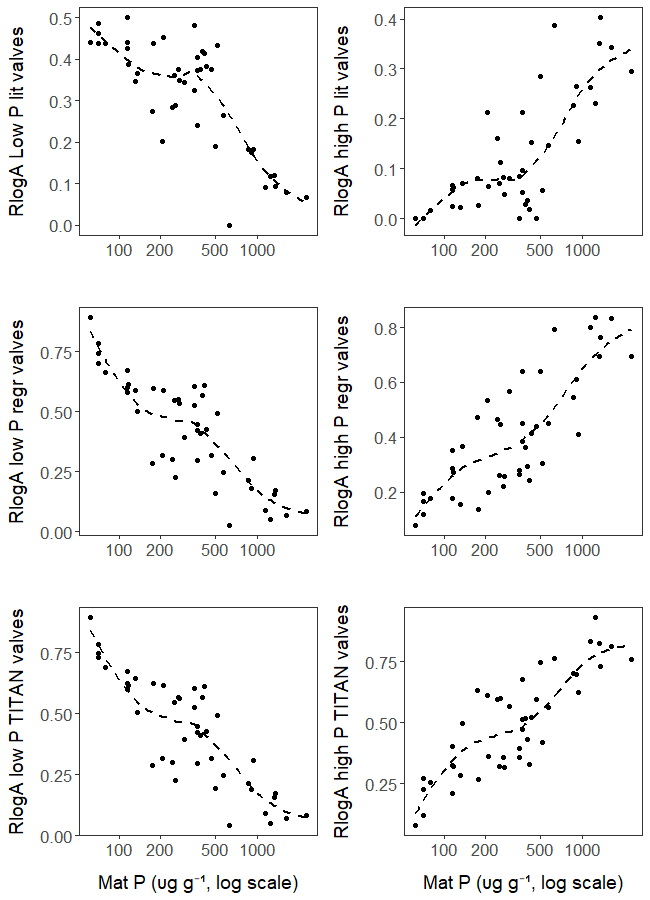


Fig. S8e. Relationships between metrics based on RlogA valves versus log-transformed mat P. The dashed black line represents a lowess fit to the data.


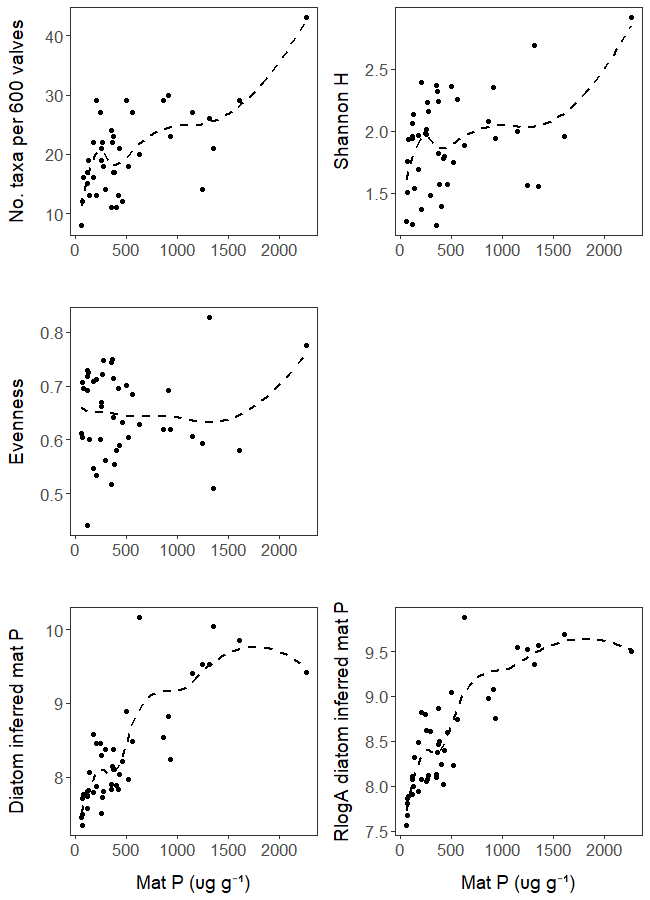


Fig. S9a. Relationships between diversity and weighted average metrics versus mat P without log transformation. The dashed black line represents a lowess fit to the data.


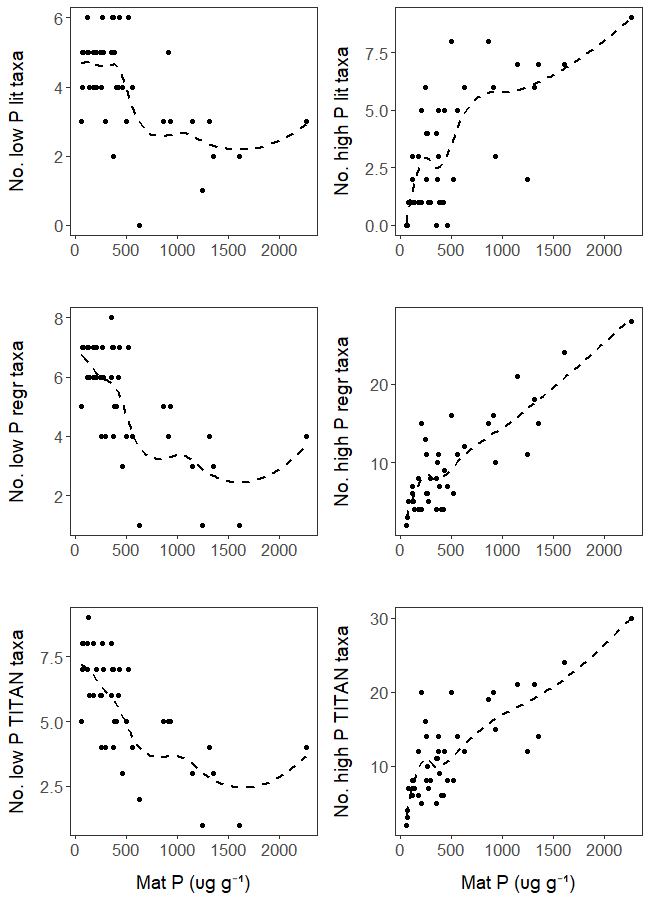


Fig. S9b. Relationships between metrics based on number of taxa versus mat P without log transformation. The dashed black line represents a lowess fit to the data.


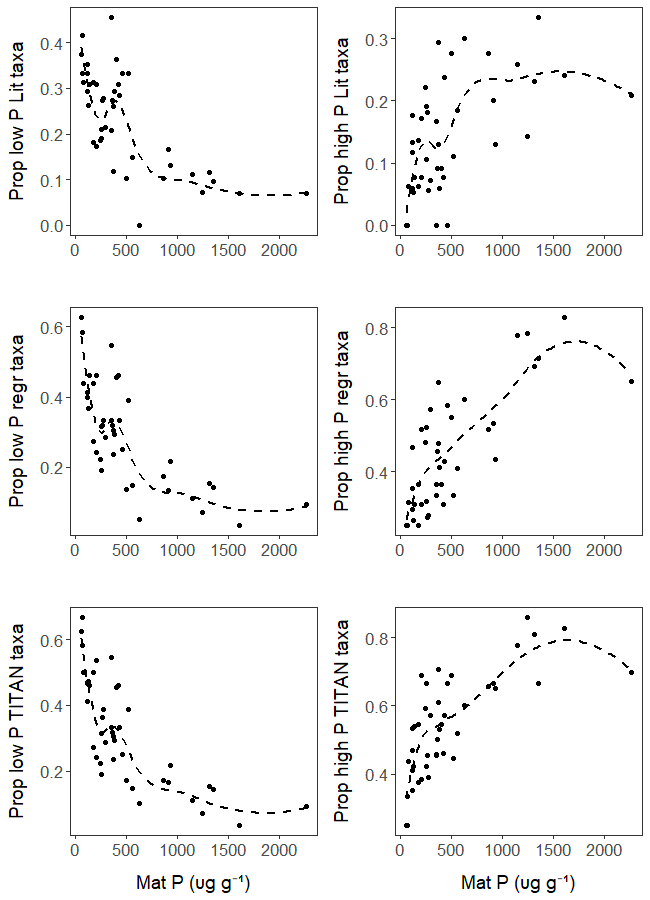


Fig. S9c. Relationships between metrics based on proportion of taxa versus mat P without log transformation. The dashed black line represents a lowess fit to the data.


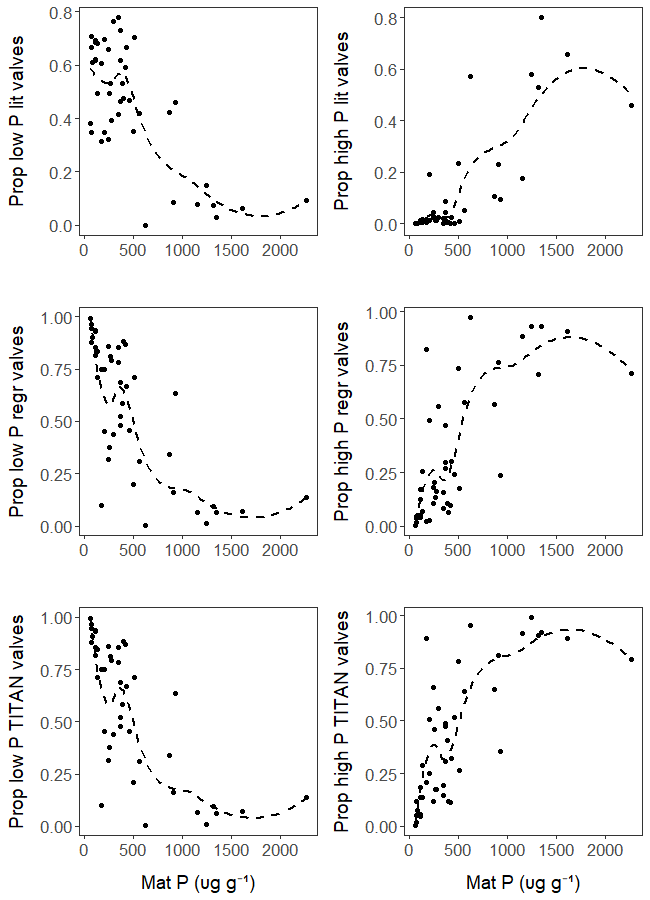


Fig. S9d. Relationships between metrics based on proportion of valves versus mat P without log transformation. The dashed black line represents a lowess fit to the data.


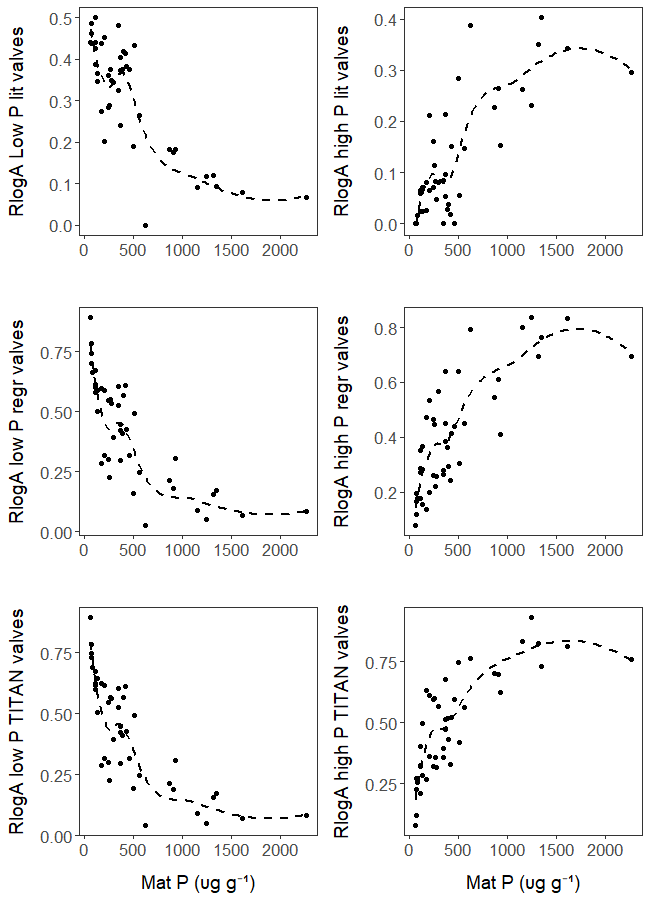


Fig. S9e. Relationships between metrics based on RlogA valves versus mat P without log transformation. The dashed black line represents a lowess fit to the data.

Table S9. Significant changepoints in metric-mat P relationships. Bold numbers indicate especially low probability of occurring by chance.

| Diversity or Trait-Based Metric | | | <100 | 100-150 | 150-250 | 250-350 | 350-450 | 450-750 | >750 |
| --- | --- | --- | --- | --- | --- | --- | --- | --- | --- |
| Trait | Source | Metric.Type |  |  |  |  |  |  |  |
| High P | TITAN | noTaxa | 71.000 | **137.000** |  |  |  | 464, **627** | **1246** |
| High P | TITAN | PropTaxa |  |  |  |  |  |  |  |
| High P | TITAN | PropValves |  |  |  |  |  |  |  |
| High P | TITAN | RlogValves |  |  |  |  |  |  |  |
| Low P | TITAN | noTaxa |  |  |  |  | 427 |  |  |
| Low P | TITAN | PropTaxa |  |  |  |  |  |  |  |
| Low P | TITAN | PropValves |  |  |  |  |  |  |  |
| Low P | TITAN | RlogValves |  |  |  |  |  |  |  |
| evenness | | diversity |  |  |  |  |  |  |  |
| richness | | diversity | 71.000 | 137.000 |  |  |  | 464,627 | 1246 |
| ShannonH | | diversity |  |  |  |  |  |  |  |
| WAM_matP | | Inferred mat P |  |  |  |  |  | 564 |  |
| WAM_RlogA.matP | | Inferred mat P |  |  |  |  |  |  |  |


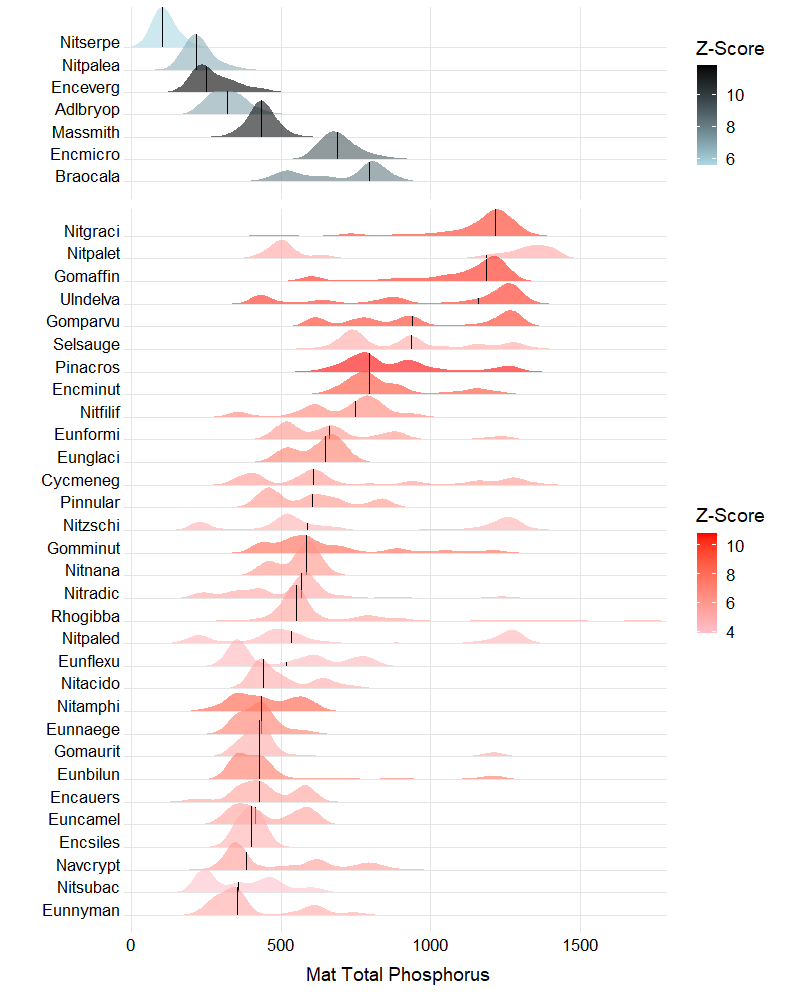


Fig. S10. Frequency distributions of likely change points for low P (gray) and high P (red) taxa determined by TITAN. Shading indicates TITAN Z-scores. Metric codes are based on the first three letters of the genus name and first five letters of the species names, which can be found in Table S3.
